# Social cognitive function in stroke survivors: A scoping review

**DOI:** 10.1016/j.cccb.2025.100398

**Published:** 2025-09-22

**Authors:** Ana Davlasheridze, Lena Rafsten, David Krabbe, Farzaneh Badinlou, Renate Reniers, Terence J Quinn, Tamar Abzhandadze

**Affiliations:** a Rehabilitation Medicine, Department of Clinical Neuroscience, Institute of Neuroscience and Physiology, Vita Stråket 12, Floor 4, University of Gothenburg, 413 45 Gothenburg, Sweden; b Department of Occupational Therapy and Physiotherapy, Sahlgrenska University Hospital, Göteborgsvägen 31, Hus S, Paviljongen 431 80 Mölndal, Sweden; c Department of Rehabilitation Medicine, Sahlgrenska University Hospital, Tunnlandsgatan 9, 421 37 Gothenburg, Sweden; d Centre for Psychiatry Research, Department of Clinical Neuroscience, Karolinska Institutet, & Stockholm Health Care Services, Region Stockholm, Norra Stationsgatan 69, Plan 7, 113 64 Stockholm, Sweden; e Birmingham Medical School, School of Medical Sciences, College of Medicine and Health, University of Birmingham, Birmingham B15 2TT, United Kingdom; f Institute for Mental Health, University of Birmingham, Birmingham B15 2TT, United Kingdom; g Centre for Human Brain Health, University of Birmingham, Birmingham B15 2TT, United Kingdom; h School of Cardiovascular and Metabolic Health, University of Glasgow, 126 University Place Glasgow, G12 8TA Glasgow, United Kingdom

**Keywords:** Theory of mind, Empathy, Emotion, Perception, Recognition, Regulation, Stroke

## Abstract

**Objective:**

We aimed to summarize current evidence on social cognitive function in stroke survivors by: identifying social cognition domains assessed; describing assessment tools; reporting impairment prevalence; and outlining interventions targeting impaired social cognition.

**Methods:**

Updated methodological scoping review guidelines were followed. Database searches were conducted by a librarian on February 26, 2024 (starting from January 1, 2000), across multidisciplinary, international databases. Two authors who independently screened title, abstract, and full texts, resolved discrepancies through discussion or senior reviewer input. Standardized data extraction forms were used, and data were analyzed using descriptive statistics.

**Results:**

Of 29,069 records, 62 studies, mostly hospital-based (52 %), using case-control designs (37 %), met the inclusion criteria. They included 3152 participants with stroke (62 % male), mainly with ischemic stroke (61 %), and right hemisphere lesions (39 %). The five domains (number, most common assessment instruments, and prevalence/or mean ± *s*.d.) of social cognitive function were: emotion perception and recognition (*n* = 38, “Ekman 60-Faces Test,” 7–100 %); theory of mind (*n* = 23, “Reading the Mind in the Eyes,” 15–64 %); empathy (*n* = 15, 24 %, “Balanced Emotional Empathy Scale,” 6–58 %); emotion regulation (*n* = 1, 2 %, “Difficulties in Emotion Regulation Scale,” range: 8.8 ± 3.87–72.07±21.54); and social problem-solving (*n* = 2, “Social Problem-Solving Fluency Task,” range: 18.02±4.62–98.1 ± 4.0). No study focused on interventions.

**Conclusion:**

Social cognition after stroke research has mainly addressed emotion recognition, theory of mind, and empathy, with limited attention to other aspects. Although social cognition impairments were common, no studies have specifically targeted their rehabilitation, underscoring the need for focused interventions.

## Highlights

• This review included 62 studies on social cognition in stroke.
• Emotion recognition is most studied in stroke survivors.
• The Ekman 60-Faces Test is the most applied assessment tool.
• Prevalence of social cognitive impairments post-stroke is wide-ranging.
• No studies identified interventions targeting social cognition after stroke.

## 1 Introduction

Social cognition is an umbrella term encompassing a wide range of mental processes that enable humans to understand themselves and others, interact with others, and navigate social environments effectively [[1], [2], [3], [4]]. These processes include perceiving and interpreting social cues, recognizing emotions, understanding others' mental states (theory of mind [ToM]), making moral decisions, regulating emotions, and empathy. Traditionally, research focused primarily on emotion recognition, ToM, and contemporary perspectives suggests that social cognition comprises three interconnected processes: perceptual/representational (e.g., perceiving facial and bodily cues), evaluative/interpretational (e.g., emotion recognition, empathy), and regulatory (e.g., emotion regulation) [[1], [2], [3], [4], [5]]. These interconnected processes are crucial for navigating complex social interactions, such as recognizing facial expressions, interpreting body language, and comprehending social norms [[2], [3], [4]].

The neural basis of social cognition involves multiple brain structures and neural networks, and this is perhaps driven by a hierarchical integration of cognitive and affective processes, supported by distinct but overlapping neural networks [6,7]. For instance, ToM may rely on regions such as the temporoparietal junction and medial prefrontal cortex, which play key roles in individuals’ understanding of others’ beliefs. In contrast, empathy is typically associated with the activation of the anterior insula and anterior cingulate cortex, which facilitate the sharing of emotional experiences [7]. At a more foundational level, the mirror neuron system, implicated in the automatic simulation of others' actions and emotions, may serve as a neural substrate for both ToM and empathy, by enabling embodied understanding through observation and imitation [8]. Together, these processes function across multiple levels, where higher-level abstract cognition and lower-level automatic affective responses interact seamlessly to support effective social interactions [7].

Stroke causes significant disruptions in brain structures and neural networks, leading to impairments in social cognitive function [9,10]. A meta-analysis found moderate to large deficits in ToM (*r* = –.44) and social perception (*r* = –.55) in stroke survivors compared to healthy controls. These impairments can manifest in the acute, subacute, and chronic phases of stroke, with some deficits persisting for even years after the event [11]. Impaired social cognitive function can significantly impact overall functioning after stroke [11,12]. It can affect interpersonal relationships, social participation, and quality of life [12]. Patients with social cognitive deficits may struggle with understanding social cues, maintaining relationships, and reintegrating into their communities [11,12].

Despite the complexity of social cognitive functioning and growing recognition that it depends on intricate, interconnected neural systems [11], current clinical practice rarely incorporates systematic assessment of social cognition in stroke survivors. This gap persists even though impairments in social cognition are common and can significantly affect long-term behavioral outcomes, including in individuals with relatively mild strokes [11]. Moreover, existing research in this area has largely focused on isolated domains, such as ToM, emotion recognition, or empathy, despite growing evidence that stroke often results in impairments across multiple social cognitive domains [11]. To support future research and stroke care in this area, we aimed to map the current evidence on social cognitive function in stroke survivors by addressing four interrelated objectives. Specifically, our objectives were to: (I) map the social cognitive domains studied among a population with stroke, (II) identify assessment tools used to measure these domains, (III) report the prevalence of social cognitive impairments post-stroke, and (IV) identify and describe interventions targeting impaired social cognition in stroke survivors.

## 2 Methods

This scoping review, which followed established methodology [13,14], is considered the most suitable approach for this study that seeks to map broad, heterogeneous evidence across social cognitive domains in stroke survivors [15]. Moreover, we aimed to explore the extent, range, and nature of existing research, identify gaps, and inform the development of future interventions [15]. We prospectively registered the review in PROSPERO as a systematic review (CRD42024508112) but ultimately carried out a scoping review. We still followed the registered study protocol and the PRISMA-ScR guidelines. The scoping review protocol was published in the Research and Development in Sweden database (date of registration, 26/01/2024; registration number, 282170).

### 2.1 Inclusion criteria

For this scoping review, the population of interest was adult stroke survivors (aged ≥18 years) with clinically or radiologically verified ischemic stroke (IS), intracerebral hemorrhage (ICH), or subarachnoid hemorrhage (SAH). Studies were eligible regardless of the time elapsed since the index stroke, allowing for inclusion of both acute and chronic phases of stroke recovery.

The central concept of this review, *social cognition*, was defined as the mental processes involved in perceiving, vicariously experiencing, interpreting, and responding to social information and interactions. This included emotion recognition, understanding social cues and norms, empathy, perspective-taking, ToM, and social problem-solving. Eligible studies were required to examine social cognitive functioning or related domains either as a primary exposure or as an outcome measure.

Included studies could originate from any geographical location or setting, including inpatient and outpatient care, rehabilitation centers, community-based environments, and research facilities. This broad inclusion enabled a comprehensive mapping of the literature across various healthcare systems and socio-cultural contexts.

The included evidence sources were primary studies using quantitative research approaches, which best aligned with the review's objective of mapping measurable dimensions of social cognition post-stroke. Only studies published in English were included, unless at least one co-author was fluent in the language of the original publication.

### 2.2 Search strategy

To guide the literature search, appropriate search terms and relevant bibliographic databases were identified in collaboration with a librarian from the Medical Library of Sahlgrenska University Hospital, Sweden, who conducted database searches from January 1, 2000 to February 26, 2024 (the date of the search). The January 1, 2000 start date was informed by two reasons: 1) to focus on the period during which indexing terminology and database coverage showed more consistency, and (ii) capture studies most relevant to contemporary practice. Search terms for each of the two areas of interest, stroke and social cognitive function, included both the terms (e.g., social cognition), their *Medical Subject Headings* (MeSH), and other domain-related terms (e.g., social intelligence and affective empathy). Search was conducted to retrieve studies from the following databases: EMBASE (Ovid), MEDLINE (Ovid), CINAHL (EBSCO), AMED (Ovid), Cochrane library (trials and reviews), PsycINFO (Ovid), and Web of Science. Detailed information on these databases, dates on which the search was conducted in each database, as well as the full electronic search strategy, are presented in supplementary material Supplementary Tables S1 and S2, respectively. The authors of the papers included were contacted for additional data when the information of interest was unavailable in the published articles.

### 2.3 Study screening and selection

The study selection was based on pre-specified inclusion and exclusion criteria outlined in the review protocol. The screening process began with a review of titles and abstracts, where one author (TA) screened all records, and a second screening was performed by distributing eligible articles among co-authors (AD, DK, or LR). For full-text review, TA reviewed all articles, with a second assessment conducted by AD, LR, or FB. Discrepancies at any stage of screening were resolved through discussion or, when necessary, adjudication by a third reviewer (TJQ or RR). To enhance consistency and minimize bias, calibration exercises were conducted among reviewers before both the title/abstract and full-text screening stages. Reasons for exclusion of sources at the full-text stage were documented. Consistent with scoping review methodology, critical appraisal or risk of bias assessment was not conducted, as the aim was to map the available evidence rather than to evaluate study quality [14].

### 2.4 Data extraction

Data extraction was performed using standardized, predefined forms. One reviewer (AD or LR) extracted the data, while a second reviewer (TA) independently verified the accuracy and completeness of the extraction for all records. Any uncertainties or discrepancies were resolved through discussion between co-authors.

The extracted data included study characteristics (author, year of publication, country, study design, and setting); participant demographics (sex, age); and stroke-related features (stroke type, lateralization, localization, and stroke phases), as defined by Bernhardt et al. [16]).

In studies with heterogeneous participant groups, we extracted data specific to stroke and social cognition wherever possible. Studies involving the same clinical stroke survivors were included if they reported distinct outcome measures. To avoid duplication and potential bias, overlapping participants and measures were counted only once in the analysis.

### 2.5 Data analysis

For social cognitive function, its related domains, and assessment instruments, data were initially charted as reported in the original studies. Since most studies focused on specific social cognitive domains, we subsequently grouped related data by domain similarity to improve clarity and facilitate synthesis. For example, facial emotion perception and prosody were consolidated under the category “Emotion perception and recognition.” The original names of the assessment instruments were retained without modification. Quantitative data from the included studies, such as publication year, country, study design, sample size, and characteristics of stroke survivors are summarized using frequency counts and percentages (n [ %]), means and standard deviations (mean [±*s*.d.]), and medians and interquartile range (median [IQR]/Q_1_–Q_3_). These descriptive statistics are presented in tables and visualized in figures. Qualitative data, including the different social cognitive function domains and assessment instruments, were categorized and described narratively. Basic coding was applied to groups with similar concepts and key features, but no in-depth thematic synthesis or meta-analysis was conducted, as this is outside the scope of a scoping review. For intervention studies, a separate Excel file was prepared; however, no eligible studies were identified for this aim.

## 3 Results

### 3.1 Overall characteristics of the included studies

**Fig. 1:**
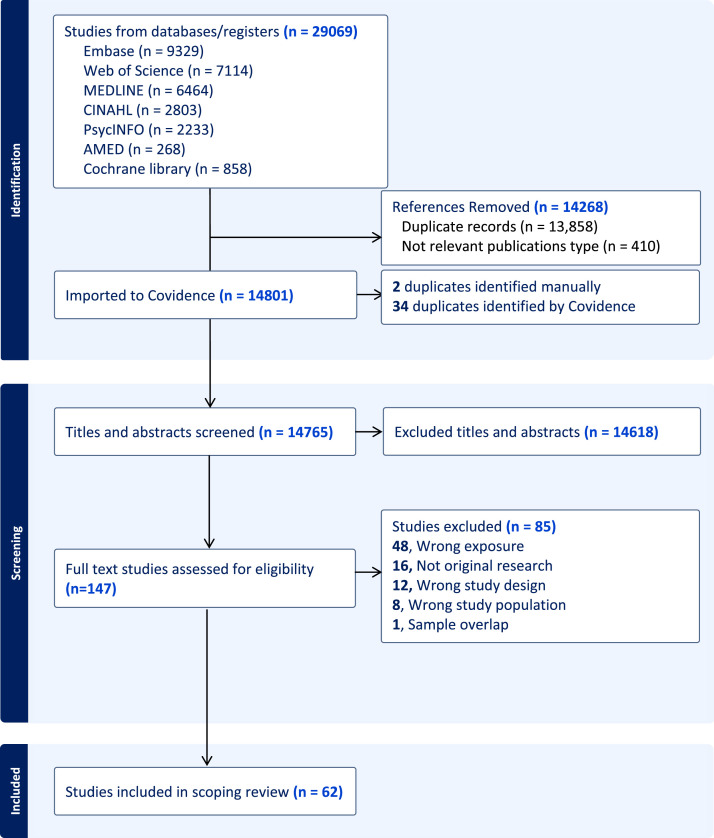
PRISMA flow diagram of the study selection in this scoping review on social cognitive function in stroke survivors.

**Figure 2:**
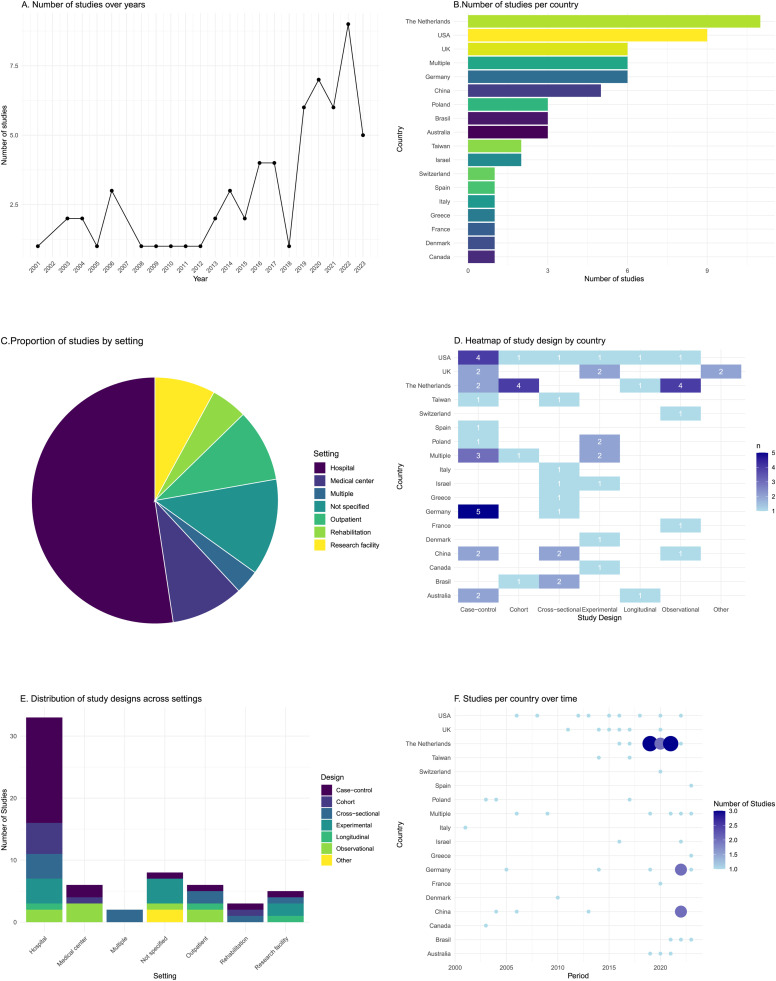
Descriptive characteristics of the studies included in the scoping review.

As Fig. 1 shows, of the 29,069 records initially identified, 62 met the inclusion criteria [[17], [18], [19], [20], [21], [22], [23], [24], [25], [26], [27], [28], [29], [30], [31], [32], [33], [34], [35], [36], [37], [38], [39], [40], [41], [42], [43], [44], [45], [46], [47], [48], [49], [50], [51], [52], [53], [54], [55], [56], [57], [58], [59], [60], [61], [62], [63], [64], [65], [66], [67], [68], [69], [70], [71], [72], [73], [74], [75], [76], [77], [78]]. In Fig. 2, >50 % of the 62 studies were published after 2019, the Netherlands had the highest number of publications (*n* = 11, 17 %), most common setting was hospitals (*n* = 33, 52 %), and most common study design was case control (*n* = 23, 37 %). Common reasons for patient exclusion were prior neurological, cognitive, or psychiatric disorders (e.g., previous stroke, dementia, depression); substance abuse history; severe cognitive impairments; language barriers (aphasia); sensory deficits; functional limitations; and practical issues such as magnetic resonance imaging contraindications or insufficient language proficiency (Supplementary Table S3). The most common inclusion criteria across studies were normal or corrected sensory functions (vision, hearing), and many studies specified unilateral lesions, particularly involving the cerebellar or supratentorial regions, right hemisphere lesions, or right-handedness (Supplementary Table S3).

The total number of stroke survivors reported in the 62 studies was 3152; 1967 (62 %) were men; phases and times since index stroke were as follows: hyperacute/acute phase (<7 days, *n* = 7, 11 %); early subacute (7 days to 3 months, *n* = 21, 34 %); late subacute (3–6 months, *n* = 3, 5 %); chronic phase (>6 months, *n* = 30, 48 %); and not applicable (one study). Most stroke survivors had IS (*n* = 1920, 61 %); 111 (4 %) had ICH; 22 (<0.5 %) had both IS and ICH; 396 (13 %) had SAH; data were missing on stroke type in 688 (22 %), whereas the remaining (*n* = 15, <0.01 %) had other conditions such as axonic damage or similar. Of the 3152 stroke survivors, 1220 (39 %), 794 (25 %), and 43 (1 %) had right-sided, left-sided, and bilateral stroke, respectively.

### 3.2 Social cognitive function in stroke survivors

**Figure 3:**
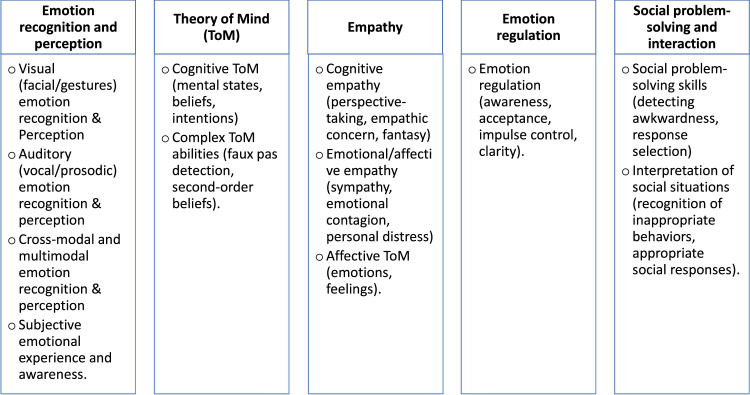
Overview of the five domains of social cognitive functions identified from the 62 included studies.

Fig. 3 shows the five social cognitive function domains, which were: (I) Emotion perception and recognition, including visual (facial/gesture), auditory (vocal/prosodic), cross-modal/multimodal emotion recognition, and subjective emotional experience; (II) ToM, encompassing cognitive, affective, and complex components; (III) Empathy, including differentiating between cognitive and emotional/affective empathy; (IV) Emotion regulation, comprising of its processes; and (V) Social problem-solving and interaction, including skills for social problem-solving and interpretation of social situations. Some studies covered several social cognitive domains, while others were specifically focused on one domain. Table 1 presents an overview of the seven most used tools for assessing emotion recognition, ToM, and empathy. The details on the format, constructs assessed, item count, administration time, and validation populations of these tools, including objective tests and self-reported measures, are shown in Table 1. The studies included were not specifically designed to enroll the stroke cohorts considering the presence of social cognitive impairment. No studies were identified that addressed interventions for social cognitive function impairment.

**Table 1:** Descriptive characteristics of instruments^a^ used to assess social cognition in stroke.

Test name	Ekman 60-Faces Test	The Faux Pas test	Reading the Mind in the Eyes Test	The Cartoon test	The Interpersonal Reactivity Index	The Balanced Emotional Empathy Scale
Construct(s) assessed	Facial emotion recognition	Theory of Mind	Theory of Mind	Theory of Mind	Dispositional empathy	Emotional/affective empathy
Task, format	To indicate which of six basic emotions best describes photographs being displayed. Paper-based or computerized.	To detect and describe faux pas situations in short stories (either self-read or read aloud by the examiner).	To indicate which of four words best describes what persons in photographs are thinking or feeling.	To interpret presented cartoons and explain a joke in it or answer questions about the characters’ intentions, beliefs, or emotions.	A self-report questionnaire measuring dispositional empathy across four dimensions (perspective taking, emphatic concern, personal distress, fantasy).	A self-report questionnaire measuring the emotional dimension of empathy.
Number of items / trials	60 photographs of human faces	10 or 20 stories, with about half containing a faux pas.	36 photographs of the eye region of faces	6–24 cartoons (usually single-panel or short comic strips)	In total 28 items (7 for each subscale) rated on a 5-point Likert scale	30 items rated on a 9-point Likert scale.
Stimulus/modality	Visual	Verbal	Visual	Visual	Written statements presented on paper or in digital format.	Written statements presented on paper or in digital format.
Population used to develop /validated on	Healthy subjects/ dementia, mental health conditions	Children with Autism Spectrum Disorders/Neurodiversity and psychiatry populations.	Healthy adults/ Neurodiversity and psychiatry populations.	Adults, children, neurotypical and various clinical groups.	College students/ validated in diverse adult and clinical samples	Healthy adults.
Validated in stroke population	Unclear	Unclear	Unclear	Unclear	Yes	No
Administration time, min	10–15	20–25	2–3	10–20	3–5	5–10
Language(s) available	English, Italian, Spanish, Korean, etc.	English, Turkish, Italian, Chinese, Portuguese, Swedish, French, etc.	English, Italian, Swedish, etc.	English, Italian, German, Spanish, etc. Cartoons may need cultural adaptation.	English, French, Chinese, Dutch, Farsi, German, Italian, Japanese, Korean, Spanish, Swedish, etc.	English
License needed	Yes	Can be free for clinical/research use.	Can be free for clinical/research use.	Can be free for clinical/research use.	No	No
Training needed	Yes	Yes, for scoring and interpretation	Yes, for scoring and interpretation	Yes, for scoring and interpretation	No	No

a Only instruments using three or more times are described.

#### 3.2.1 Emotion perception and recognition post-stroke

Emotion perception and recognition were investigated in 38 studies [[17], [18], [19],^21^,^22^,^26^,^27^,[29], [30], [31], [32], [33], [34],^37^,^41^,^42^,^44^,^46^,^48^,^49^,[51], [52], [53], [54], [55],[60], [61], [62],^64^,[66], [67], [68], [69], [70],^73^,^74^,^77^,^78^], with the majority (*n* = 22, 58 %) conducted during the chronic phase of stroke (Table 2). The most studied social cognitive subdomain was facial emotion recognition [[17], [18], [19],^22^,^26^,^27^,[29], [30], [31], [32], [33],^37^,^41^,^42^,^44^,^46^,^48^,^49^,^51^,[53], [54], [55],^60^,^62^,^64^,^70^,^73^,^74^,^77^,^78^]. The Ekman’s 60-Faces test was the most applied assessment tool [17,22,[29], [30], [31],^44^,^53^,^54^,^60^,^62^,^64^,^73^,^74^]. The overall prevalence of impairments, which were often related to the right hemisphere structures, ranged 7–100 % [22,26,32,41,52,55,61,70]. Table 2 shows that the deficits were more pronounced for negative emotions [17,18,27,60,68,73,74,77].

**Table 2:** Characteristics of studies on emotion perception and recognition in stroke survivors.

Author (year); study design	Major results related to social cognitive function	No. of patients (M: F); age mean (±SD)/median (IQR), y	Type of stroke; lateralization, n	Stroke location, n	Stoke severity, instrument name; and phase (days)	Social cognitive function domain	Name of the instrument	Prevalence of impairment, n ( %)	Unit of measurement (median/IQR, mean/±SD)
Aben [17]; Cohort	Out of the total 230 participants with stroke, 77 had impaired emotion recognition (33.5 %).	230 (154:76); 70.15 (±8.9)	IS 230; R 90, L 99	Cortical 82 Supratentorial right 90 Supratentorial left 99 Infratentorial 51	2.5 (1–4), NIHSS; Early subacute (35 ± 7)	Facial emotion recognition	Ekman’s 60-Faces Test	77 (33.5)	N/A
Adamaszek [18]; Case-control	Participants with cerebellar strokes showed impairments in recognizing emotions, especially negative ones like fear.	15 (12:3); 61.00 (±14.06)	IS 15; R 4, L 7, B 4	Cerebellum 15	N/A; Early subacute (range 7–35)	Facial identity discrimination	Tübingen Affect Battery	N/A	96.9 (±6.7)
						Non-emotional prosody discrimination			95.5 (±4.6)
						Facial affect discrimination			84.1 (±11.8)
						Facial affect naming			83.3 (±11.6)
						Facial affect selection			83.7 (±11.6)
						Facial affect matching			73.7 (±13.7)
						Emotional prosody discrimination			97.5 (6.1)
						Name the emotional prosody			66.2 (±14.7)
						Conflicting emotional prosody			68.5 (±12.5)
						Match emotional prosody to an emotional face			65.4 (±21.7)
						Match emotional face to the emotional prosody			68.9 (±22.0)
Adamaszek [19]; Case-control	Cerebellar stroke was associated with impairment in recognition and discrimination of cues of both facial and vocal expressions of differing basic emotions.	22 (17:5); 55 (±9)	IS 22; R 15, L 7	Cerebellar 22	N/A; Early subacute (58 ± 48)	Recognition and discrimination of emotional facial expression and of emotional prosody	Tübingen Affect Battery	N/A	79 (±8.5)
		16 (14:2); 56 (±8)	IS 16; N/A	Supratentorial 16	1.9 (±0.8), NIHSS; Early subacute (22 ± 6)	Recognition and discrimination of emotional facial expression and of emotional prosody			N/A
Adams [21]; Case-Control	Participants with stroke exhibited significant difficulties in emotion perception.	35 (23:12); 64.69 (±12.92)	ICH 5, IS 30; R 18, L 17	Frontal lobe 5, Parietal lobe 3, Occipital lobe 3, Cerebellum 6, Basal ganglia 1, Multi-lobe 8, Subcortical/other structures 7, N/A 2	N/A; Chronic (415.20 ± 415.20)	Emotion perception	The Awareness of Social Inference Test-Short Form		6.29 (±1.49)
						Emotion perception	Lifespan Database of Adult Emotional Facial Stimuli Database		40.17 (±5.07)
Alvarez-Fernandez [22]; Case-Control	Participants with stroke performed lower in facial emotional recognition and had a less intense subjective emotional response to social content stimuli compared to healthy subjects.	41 (21:20); 68.5(±12.2)	IS 41; R 41	Frontal lobe 10, Parietal lobe 25, Temporal lobe 22, Occipital lobe 7, Subcortical/other structures 57	N/A; Chronic (318.9 ± 147.4)	Facial emotion recognition	Ekman’s 60 Faces Test	N/A	35.3 (±11.0)
						Subjective emotional experience	International Affective Picture System	N/A	4.5 (±0.4)
Blonder [26]; Case-Control	Participants with RHD struggled with recognizing emotions through facial and prosodic expressions and report higher negative affect compared to controls.	12 (8:4); 55.8 (±13)	IS 12; R 12	N/A	N/A; Early subacute (32 ± 15)	Facial identity discrimination	Florida Affect Battery results	N/A	82.9 (±16.6)
						Facial affect discrimination			75 (±15.1)
						Facial affect naming			76.7 (±17.6)
						Facial affect selection			79.6 (±19)
						Facial affect matching			63.8 (±25.8)
						Nonaffective Prosody Discrimination			81.3 (±15.8)
						Affective Prosody Discrimination			90 (±12.4)
						Name affective Prosody			69.2 (±21.1)
						Cross-modal Matching Prosody to face			69.6 (±23)
						Cross-modal Matching face to prosody			74.1 (±21.9)
Braun [27]; Case-control	Participants with stroke showed significantly worse emotion recognition compared to healthy controls, especially for negative emotions.	24 (12:12); 65.4 (±13.6)	IS, ICH (N/A); R 13, L 11	Multi-lobe 19, Occipital lobe 1, Frontal lobe 1, Parietal lobe 1, N/A 2	50.2 (±7.7), SSS; Early subacute (26 ± 5)	Facial emotion recognition	Facially Expressed Emotion Labelling	N/A	23.5 (±6.8)
Cheung [33]; Case-control	Participants with subcortical stroke had difficulty recognizing facial emotion.	38 (28:10); 64.89 (±10.33)	N/A; R 19, L 19	LSS 19, RSS 19	N/A; Early subacute (7–121)	Facial emotion recognition	Interpolated Emotional Expressions Test by Wang	N/A	RSS 42.11 (±9.98) LSS 45.26 (±11.58)
Cooper [34]; Exploratory	Participants with stroke showed significantly impaired emotion perception compared to the control group.	28 (17: 11); 67.75 (±9.18)	IS 28; R 10, L 12, B 6	N/A	N/A; Chronic (413 ± 30.98)	Facial emotion perception	Facial Expressions of Emotion Stimuli and Test	N/A	1.93(±14.82)
						Auditory emotion perception	A sub-test of the Florida Affect Battery		68.15 (±17.49)
						Multi-modal emotion perception	The Awareness of Social Inference Test		65.18 (±19.51)
De Souza [37]; Cross-sectional	Specific emotional recognition patterns and cognitive associations were observed in participants with stroke that were not present in the control group.	18 (14:4); 62.89 (±11.74)	IS 18; N/A	N/A	3.56 (±2.00), NIHSS: Early subacute (range 40–60)	Facial emotion recognition	Facial Emotion Recognition Test	N/A	24.5 (22.00–27.25)
Harciarek [41]; Case-control	The RHD regardless of lesion location, lead to an impairment in recognizing emotional faces. The patients with ASRH, in comparison to individuals with PSRH, were more impaired at recognizing emotional facial expressions.	27 (14:13); 63.97 (±10.07)	IS 27; R 27	Frontal 4, Fronto-temporal 3, Subcortical 5, Temporo-parietal 8, Temporo-occipital 2, Parieto-occipital 2, Parietal 3.	N/A; Chronic (265 + 113.85)	Facial emotion recognition	Pictures of Facial Affect	N/A	ASRH 11.82 (±2.86) PSRH 16.94 (±2.41)
Harciarek [42]; Case-control	Participants with stroke scored significantly lower than normal subjects on emotional prosody and facial affect recognition tests. No differences were found between RHD patients with and without hemispatial neglect. A significant correlation between emotional perception of faces and prosody was found in the RHD group.	30 (14:16); 63.83 (±10.15)	IS 30; R 30	Frontal 2, Fronto-temporo-parietal 2, Temporo-parietal 10, Subcortical 5, Unknown 4, Temporo-occipital 2, Parieto-occipital 2, Parietal 3.	N/A; Chronic (ca 306)	Prosody	Emotional Prosody Test	30 (100)	8.41 (±2.69)
						Facial emotion recognition	Facial Affect Recognition Test	30 (100)	9.56 (±2.46)
Jorna [44]; Observational	No differences between SAH and TBI participants was found in terms of emotion recognition, negative emotion recognition, and anger misattribution.	80 (30:50); 55.8 (±10.0)	SAH 80; N/A	N/A	Low 66 (82) %, WFNS ≤ 3; Chronic (1022.7 ± 219)	Facial emotion recognition	The Ekman’s 60 Faces Test	N/A	46.3 (±6.9)
						Negative emotion recognition			27.8 (±6.2)

Kucharska-Pietura [48], Experimental	The results confirm the primacy of the right hemisphere in processing all emotional expressions across modalities—both positive and negative—but suggest that left hemisphere emotion processing is modality-specific.	60 (35:25); 56.35 (±9.65)	IS 53, ICH 6; R 30, L 30	Frontal 12, Parietal 15, Frontoparietal 7, Frontotemporal 9, Temporal 10, Occipital 4, Globus pallidus 2, Thalamus 1.	N/A; Early subacute (≥28)	Facial emotion labelling	A set of 36-photographs	N/A	RHD 43.3 (±15.9) LHD 68.4 (±9.0)
						Facial emotion recognition	A set of 36-photographs		RHD 48.3 (±15.0) LHD 72.9 (±8.8)
						Prosody	A series of five semantically neutral sentences		RHD 39.9 (±16.6) LHD 54.6 (±16.4)
Luo [51]; Cross-sectional	Facial emotion perception and recognition deficits may occur in participants with acute ischemic stroke and are associated with impaired cognitive functions, where the cerebral hemispheres and the infratentorial brain are jointly involved.	47 (20:27); LBD: 70.50(±10.71) RBD: 60.20(±12.80) IBD: 58.77(±11.72)	IS 47, R 20, L 14	Hemispheres 34, Infratentorial 13	NIHSS: LBD 3.76 (±1.42), RBD 3.85 (±2.23), IBD 4.15(±2.12): Acute (LBD 8.00 ± 1.18; RBD 8.20 ± 1.24; IBD 8.38 ± 1.19)	Facial Emotion perception and recognition	The Southeastern China Brief Affect Recognition Test	N/A	LBD 14.29 (±1.03), RBD 16.71 (±0.83), IBD 15.47 (±1.04)
Nijsse [53]; Inception cohort	Social cognition tests showed significant correlations with each other and with tests for visual perception, language, mental speed, and cognitive flexibility.	148 (103:45); 63.9 (±11.3)	IS 137, ICH 11, R 62, L 50	Hemispheres 112 Vertebrobasilar 36	2.0 (±3.0), NIHSS; Chronic (range 1095–1462)	Facial emotion recognition	The Ekman’s 60-Faces Test	N/A	42.63 (±6.2)
Nijsse [54], Cohort	Participants with stroke performed significantly worse on emotion recognition than controls.	119 (85:34); 64.3 (±11.0)	IS 111, ICH 8; R 50, L 38	Hemispheres 88 Vertebrobasilar 31	2.0 (±3.1), NIHSS; Chronic (range 1095–1462)	Facial emotion recognition	The Ekman’s 60-Faces Test	N/A	42.66 (±6.2)
Sensenbrenner [60]; Observational	Social cognition disorders were frequent 3 years after a first-ever stroke. Facial emotion recognition was particularly difficult for fear, sadness, and anger.	43 (26:23); 66.8 (±15.1)	IS 38, ICH 5, R 13, L 16	Cerebral hemisphere 29, Brainstem or cerebellum 14	N/A; Chronic (1168 ± 73.05)	Facial emotion recognition	The Ekman’s 60-Faces Test	20 (46)	N/A
Sheppard [61]; Observational	The posterior superior temporal gyrus in the right hemisphere ventral stream was critical for emotion identification in speech.	23 (12:11); 55.09 (±17.22)	IS 23, R 23	Middle cerebral artery 18, Posterior cerebral artery 3, Lenticulostriate stroke 1, Multiple locations 1	N/A; Acute (5)	Emotional prosody identification	Listening to 24 prerecorded semantically neutral sentences	16 (69.53)	N/A
Smith-Spijkerboer [62]; Observational	Impaired visual emotion recognition was found in about one-quarter of patients with minor ischemic stroke.	112 (70:42); 70.6 (±10.15)	IS 112; R 41, L 44	Cortical 41, Subcortical 40, Lacunar 30, Posterior circulation 3	≤3, NIHSS; Early subacute (42)	Visual emotion recognition	The Ekman’s 60-Faces Test	28 (25)	N/A
Stiekema [64]; Longitudinal	Participants with a minor stroke had mild impairments in emotion recognition.	118 (85:33); 67.4 (±11.1)	IS 109, ICH 9; R 54, L 38	Cerebral hemisphere 92, Vertebrobasilar 23	2.0 (±3.0), NIHSS; Chronic (range 1096–1461)	Facial emotion recognition	The Ekman’s 60-Faces Test	N/A	43.0 (±6.2)
Thomasson [66]; Experimental	Patients with right-hemispheric stroke struggled to recognize neutral or negative vocal emotions. These difficulties were linked to brain lesions in specific areas.	24 (13:11); 63.42 (±11.68)	IS 24; R 13, L 11	Cerebellum 24 (Lobules only 3, lobules and crus 12, lobules and vermis 1, multiple 7)	N/A; Chronic (889 ± 1035)	Vocal emotion recognition	A set of 60 vocal stimuli extracted from a validated database consisting of meaningless speech	N/A	N/A
Thomasson [69]; Case-control	Patients with stroke and healthy people reacted differently to scary sounds. Stroke patients made mistakes in judging how surprising the sounds were. These mistakes were linked to specific parts of the brain.	15 (9:6): 62.8 (±9.31)	IS 15; R 8, L 7	Cerebellar stroke 8, Cerebral hemisphere stroke 7	N/A; Chronic (N/A)	Vocal emotion recognition	A set of 60 vocal stimuli extracted from a validated database consisting of meaningless speech	N/A	N/A
Tippett [70]; Case-control	Damage to the right amygdala and anterior insula significantly impaired the recognition of angry and happy faces.	30 (17:13); 52.8(±12.1)	IS 30; R 30	Amygdala 7, Anterior insula 11	N/A; Acute (N/A)	Facial emotion recognition: positive	Facial Expression Task	2 (7)	N/A
						Facial emotion recognition: negative		15 (50)	
						Facial emotion recognition: neutral		5 (18)	
Van den Berg [73]; Case-control	Participants with cerebellar stroke struggled more than healthy controls to recognize emotions, especially disgust, fear, sadness, and surprise. Brain scans showed that specific brain regions were linked to these emotions.	110 (76:34); 58.5 (±11.7)	IS 110; R 48, L 44, B 18	Cortical areas of cerebrum 110	N/A; Chronic (629 ± 105)	Facial emotion recognition, total	The Ekman’s 60-Faces Test	N/A	42.9 (±7.4)
						Anger			7.5 (±2.1)
						Disgust			6.1 (±2.5)
						Fear			5.3 (±2.4)
						Happiness			9.6 (±0.9)
						Sadness			6.1 (±2.3)
						Surprise			8.4 (±1.7)
Van den Berg [74]; Case-control	Participants with stroke, compared to healthy controls, had significantly more difficulty recognizing emotional expressions and took more risks. Additionally, a poorer ability to recognize fearful facial expressions was strongly linked to an increase in risky decisions	13 (11:2); 61.4(±10.9)	IS 13; R 4, L 6, B 3	Cerebellar 13	N/A; Early subacute-chronic (range 30–1200	Facial emotion recognition, total	The Ekman’s 60-Faces Test	N/A	37.2 (±7.2)
						Anger			6.4 (±2.1)
						Disgust			5.2 (±3.1)
						Fear			3.9 (±1.8)
						Happiness			9.7 (±0.6)
						Sadness			4.8 (±2.2)
						Surprise			7.5 (±2.7)
Yeh [77]; Case-control	Participants with stroke had specific deficits in recognizing surprised and disgusted facial expressions compared to controls, but general emotions remained largely unaffected by these impairments.	34 (15:17); 60.82 (±11.21)	N/A; R 20, L 14	Basal ganglia 12, Frontal 5, Thalamus 6, Pons 6, Parietal 2, Frontoparietal 1, Occipitoparietal 1, Subcortical 1	N/A; Chronic (range 553–622)	*Facial emotion recognition,* Sad	A computerized task for emotional face recognition	N/A	Right: 3.05 (±1.64) Left: 2.57 (±1.91)
						Happy			Right: 4.55 (±0.61) Left: 4.43 (±1.60)
						Surprise			Right: 3.65 (±1.79) Left: 3.50 (±2.03)
						Angry			Right: 2.15 (±1.46) Left: 2.07 (±1.21)
						Fear			Right: 1.35 (±1.14) Left: 1.29 (±1.38)
						Disgust			Right: 2.20 (±1.91) Left: 2.50 (±1.74)
						Neutral			Right: 4.10 (±1.45) Left: 4.36 (±1.91)
Yip [78]; Cros-sectional	Participants with sub-cortical stroke were generally impaired on emotion recognition, except for facial emotion discrimination and tasks involving happy expressions.	14 (10:4); 65.29 (±9.45)	N/A; R 6, L 8	Sub-cortical 5, Thalamus 2, Pons 2, Internal capsule 4, Basal ganglia-internal capsule 1.	N/A; Chronic (254.76 ± 31.66)	(Facial) Emotion Recognition, overall	Japanese and Caucasian Facial Expressions of Emotion	N/A	85.85 (±15.05)
						Facial emotion recognition			32.14 (±4.79)
						Facial emotion identification			6.29 (±2.13)
						Facial emotion discrimination			25.86 (±3.01)
						Prosodic emotion recognition	Prosodic Emotion Recognition: identification and discrimination task		53.38 (±10.52)
						Prosodic emotion identification			9.79 (±2.99)
						Prosodic emotion discrimination			43.31 (±8.05)
Buunk [29]; Prospective cohort	Participants with aneurysmal subarachnoid hemorrhage (aSAH) were clearly impaired in emotion recognition compared to healthy individuals. However, patients with angiogram-negative subarachnoid hemorrhage (anSAH) did not perform significantly better than aSAH patients in this area.	119 (77:42): Mean 53.35	aSAH 90, anSAH 29; N/A	Anterior 69, Posterior 21	29 (24) WFNS score 4–5/high; Early subacute-Chronic (60–365)	Facial emotion recognition, total	The Ekman’s 60-Faces Test	aSAH 27 (30) anSAH 5 (17.2)	aSAH 45.64 (±6.76) anSAH 6.21 (±6.63)
						Anger		aSAH 6 (6.7) anSAH- 3 (10.3)	aSAH 7.55 (±2.02) anSAH 7.38 (±1.99)
						Disgust		aSAH 15 (16.7) anSAH 4 (13.8)	aSAH 7.13 (±2.17) anSAH 7.72 (±2.04)
						Fear		aSAH 15 (16.7) anSAH 6 (20.7)	aSAH 5.51 (±2.19) anSAH 5.52 (±2.64)
						Happiness		aSAH 1(1.1) anSAH 0(0)	aSAH 9.84 (±0.40) anSAH 9.90 (±0.31)
						Sadness		aSAH 23 (25.6) anSAH 6 (20.7)	aSAH 6.69 (±2.13) anSAH 7.03 (±2.24)
						Surprise		aSAH 2 (2.2) anSAH 1 (3.4)	aSAH 8.80 (±1.34) anSAH 8.66 (±1.32)
Buunk [30]; Observational	There was no significant difference in social cognition between SAH participants who had complete return to work and those with incomplete.	71 (43:28); 43.2 (±7.9)	SAH 71; N/A	Aneurysm circulation anterior 63, Posterior 8	11 (24) WFNS score 4–5/high; Late subacute (140.0 ± 42.6)	Facial emotion recognition	The Ekman’ 60-Faces Test	N/A	46.3 (±6.5)
Buunk [31]; Observational	Participants with aneurysmal subarachnoid hemorrhage (aSAH) showed significant impairments in emotion recognition compared to healthy controls. However, these social cognitive deficits were not associated with specific brain lesions.	88 (27:61); 53.3 (±9.8)	SAH 88; N/A	Aneurysm circulation anterior 69, Posterior 19	18 (20.5 %) WFNS score 4–5/high; Late subacute (143.07 ± 54.79)	Emotion recognition	The Ekman’ 60-Faces Test (subset)	N/A	45.7 (±6.7)
Kuttenreich [49]; Cros-sectional	Participants with stroke-related central facial paresis had significantly lower average accurate emotion recognition abilities with respect to the facial modality compared to the auditory modality.	63 (38:25); Range 35–83	IS 48, ICH 11, Both 2; R 19, L 27, B 2	N/A	N/A; Subacute (range 1359–1558)	Visual Facial Emotion Recognition, Accuracy in %	The My face training Program	N/A	FP 27.77 (±11.04) NFP 40.79 (±15.59)
						Visual Facial Emotion Recognition, time in seconds			FP 3.14 (±0.47) NFP 3.19 (± 0.34)
						Auditory Emotion Recognition, Accuracy in %	A sub-portion of the Montreal Affective Voices		FP 46.23 (±11.63) NFP 48.05 (± 11.78)
						Auditory Emotion Recognition, Time in seconds			FP 3.20 (±1.20) NFP 48.05 (± 0.88)
						Subjective Facial Emotion Recognition, accuracy	The Self-Assessment Questionnaires Emotion Recognition Accuracy and Time		FP −0.71 (±1.90) NFP −0.03 (± 1.32)
						Subjective Facial Emotion Recognition, time			FP −1.91 (± 2.90) NFP −1.00 (± 2.52)
Klepzig [46]; Case-control	Participants with stroke struggled more with recognizing emotions, especially happy expressions, compared to healthy controls. Regarding the response times, fearful expressions were recognized more slowly than happy and neutral ones.	29 (20:9); 64.2 (±13.5)	IS and ICH (N/A); R 11, L 15, B 1	Hemispheres 27, Pons 1, Cerebellum 1	4 (median), NIHSS; Chronic (1038 ± 1087)	Emotion recognition (accuracy %), fear	Emotion Recognition Task	N/A	83.0 (±16.4)
						Anger			83.0 (±16.4)
						Disgust			77.0 (±18.7)
						Happiness			98.0 (±5.3)
						Neutral			82.8 (±19.4)
O'Connell [55]; Case-control	Right-hemisphere stroke participants struggled with emotion recognition, performed worse than controls even after accounting for minor mistakes.	18 (11:7); 62.3 (±9.4)	IS 18; R 18	MCA territory 18, Pons 4	3.9 (±2.8), NIHSS; Chronic (572.3 ± 353.1)	Emotion Recognition, accuracy %	Geneva Emotion Recognition Test – Short	N/A	38.3(±17.3)
						Positive emotion target, accuracy %			47.5 (±20.4)
						Negative target trials, accuracy %			32.9 (±19.6)
Charbonneau [32]; Experimental	RBD participants performed worse than LBD patients and controls in emotion recognition. They struggled with identifying and producing emotions like surprise, happiness, fear, sadness, and anger. LBD patients generally performed as well as controls on most tasks.	32 (20:12); 61.96 (±6.03)	IS 32; R 15, L 17	N/A	N/A; Chronic (1013 ± 446)	Facial emotions: Sadness	Slides of the six usual transcultural emotions (Ekman's test)	N/A	LBD 6.69 (±1.16) RBD 6.45 (±1.46)
						Happiness			LBD 7.39 (±0.80) RBD 6.99 (±1.13)
						Surprise			LBD 6.79 (±0.81) RBD 6.13 (±0.96)
						Anger			LBD 5.49 (±1.61) RBD 5.18 (±1.40)
						Fear			LBD 4.92 (±1.55) RBD 4.42 (±1.40)
						Disgust			LBD 6.44 (±1.16) RBD 6.27 (±1.38)
						Emotional prosody: Sadness	Listening to recorded sentences (presented to the subjects via a tape recorder)		LBD 6.82 (±0.71) RBD 5.89 (±0.91)
						Happiness			LBD 5.80 (±1.26) RBD 5.89 (±1.20)
						Surprise			LBD 5.79 (±1.13) RBD 6.00 (±1.16)
						Anger			LBD 6.16 (±1.04) RBD 6.16 (±0.98)
						Fear			LBD 4.65 (±1.53) RBD 3.42 (±1.07)
						Disgust			LBD 4.24 (±1.08) RBD 3.69 (±1.14)
Nakhutina [52]; Longitudinal	RBDs and LBDs showed impairments at baseline compared to NCs. While LBDs tended to improve over time, RBDs significantly declined. Frontal lesions negatively impacted prosodic emotional expression in RBDs, but lesion extent did not systematically affect performance. No significant relationships were found between prosodic emotional perception and expression tasks.	17 (9:8); 65.3 (±10.9)	N/A; R 8, L 9	Frontal 8, Temporal 7, Parietal 6, Occipital 2, Corona radiata 4, Internal/external capsule 9, Optic radiations 1, Basal ganglia 2, Caudate 2, Putamen 1, Claustrum 2, Thalamus 1	N/A; Early subacute – Chronic (range 60.88–491.56)	Posed prosodic emotional expression	The Prosodic Expression to Command task	N/A	N/A
						Prosodic expression	The Intonation Contours task	N/A	N/A
Thomasson [67]; Cohort	The cerebellum and basal ganglia have complementary roles in emotional prosody decoding, with possible hemispheric specialization.	24 (N/A); 61.9 (±11.24)	IS 24; R 13, L 11	Cerebellum 24	N/A; Chronic (909.26 ± 128.29)	Emotional prosody recognition	Participants listen to meaningless speech (60 pseudowords) expressed in five different emotional prosodies.	N/A	N/A
Thomasson [68]; Experimental	Participants with cerebellar strokes struggle more with emotion recognition compared to healthy controls, especially with emotions such as anger and sadness.	27 (16:11); 57.63 (±11.37)	IS 27; R 16, L 11	Cerebellum 27	N/A; Chronic (812.75 ± 1002.39)	Vocal emotion recognition	60 pseudowords pronounced by 12 different actors each in 1 of 5 different prosodies.	N/A	N/A

**Abbreviations:** M, male; F, female; SD, standard deviation; IQR, interquartile range; n, number; R, right; L, left; B, bilateral; NIHSS, National Institutes of Health Stroke Scale; ICH, intracerebral hemorrhage; IS, ischemic stroke; ASRH, acute stroke with right hemispheric involvement; PSRH, post-acute stroke with right hemisphere involvement; SAH, subarachnoid hemorrhage; aSAH, aneurysmal subarachnoid hemorrhage; anSAH, angiogram-negative subarachnoid hemorrhage; WFNS, World Federation of Neurosurgical Societies; SSS, Scandinavian Stroke Scale; LBD, left brain-damaged; RBD, right brain-damaged; IBD, infratentorial brain-damaged; MCA, middle cerebral artery; NCs, normal controls; FP, facial paresis; nFS, non FP.

#### 3.2.2 ToM

ToM was examined in 23 studies [20,21,[23], [24], [25],^28^,^31^,^36^,[38], [39], [40],^43^,^53^,^54^,^58^,^60^,^64^,^65^,^71^,^72^,[75], [76], [77]], with most (*n* = 18, 48 %) conducted during the chronic phase of stroke (Table 3). Most studies did not distinguish between various subdomains of ToM. Commonly applied assessment instruments were the Reading the Mind in the Eyes task [20,21,28,38,40]; Faux Pas Test (belief attribution) [31,53,54,60,76,77]; and Cartoon Test [24,31,53,54,64]. The prevalence of impairment ranged 15–64 % (Table 3).

**Table 3:** Characteristics of studies on Theory of Mind (ToM) in stroke survivors.

Author (year); design	Major results related to social cognitive function	No. of patients (M: F); age mean (±SD)/median (IQR), year	Type of stroke; lateralization, n	Stroke location, n	Stoke severity, instrument name; and phase (days)	Social cognitive function domain	Name of the instrument	Prevalence of impairment, n ( %)	Unit of measurement (median/IQR, mean/±SD)
Adams [20]; Longitudinal	Baseline difficulties in ToM were linked to self-reported depression and loneliness.	53 (34:19); 63.9 (±15.03)	ICH 4, IS 44, NA 5; R 23, L 29	Frontal lobe 5, Parietal lobe 2, Temporal lobe 1, Occipital lobe 3, Multi-lobe/general Cortical 1, Watershed area 1, Basal Ganglia/internal capsule 11, White matter 3, Brainstem 2, Cerebellum 5, MCA territory 15, PCA territory 2, PICA territory 1, NA 4	N/A; Acute (5.1 ± 4.83)	ToM	The Reading the Mind in the Eyes Test.	N/A	22.02 (±5.5)
Adams [21]; Case-Control	Participants with stroke exhibited significant difficulties in ToM.	35 (23:12); 64.69 (±12.92)	ICH 5, IS 30; R 18, L 17	Frontal lobe 5, Parietal lobe 3, Occipital lobe 3, Cerebellum 6, Basal ganglia 1, Multi-lobe 8, Subcortical/other structures 7, N/A 2	N/A; Chronic (415.20 ± 415.20)	ToM	Reading the Mind in the Eyes Test	N/A	24.52 (±4.62)
					
					
Besharati [25]; Experimental	Perspective taking (mental but not visuospatial), rather than mentalization was the critical deficit in this population (in the sense that they could perform the ToM task in the first-person perspective, and they passed easier false belief tasks).	30 (14:16); 68.44 (±12.73)	N/A; R 30	N/A	N/A: Early subacute (8.5 IQR 9.5)	ToM	ToM stories	N/A	AHP Median 56.25 HP Median 75
Burke [28]; Case-control	Patients with aneurysmal subarachnoid hemorrhage exhibited deficits compared to control subjects.	38 (25:13); 58.47 (±10.32)	aSAH 38; N/A	N/A	N/A; Chronic (400 ± 119)	ToM	Reading the Mind in the Eyes Test	6 (15.5)	
Corradi-Dell’Acqua [36]; Observational	Impairments in ToM were linked to dysfunctions in specific brain networks rather than direct damage to isolated regions. Difficulties in inferring others' beliefs were associated with dysfunction in right prefrontal areas, while emotion understanding was linked to the left anterior insula.	40 (24:16); 61.5 (53–72)	N/A; R 25, L 15	N/A	N/A; Late subacute (195.57, range 36.75–350.40)	ToM	A modified version of the ToM paradigm	14 (35)	N/A
Dominguez [38]; Mixed methods	Participants with stroke showed deficits in ToM, specifically on the Reading the Mind in the Eyes Test.	64 (42:22); 62.16(±15.74)	ICH 9, IS 55; N/A	N/A	N/A; Acute (6.35 ± 5.82)	ToM	The Reading the Mind in the Eyes Test	22 (34.38)	61.50 (±13.90)
Ferreira Pereira [39]; Cross-sectional	The ToM Task Battery was a reliable tool for assessing social cognition in post-stroke patients.	38 (20:18); 62.68 (±13.53)	ICH 8, IS 30; R 15, L 23	N/A	N/A; Chronic (1019.74, range 350.06–1735.08)	ToM	The ToM Task Battery		4 (3–7)
Hamilton [40]; Case-control study	A right hemisphere stroke impairs ToM ability, while a left hemisphere stroke does not. Stroke had no effect on a similar non-ToM task. High correlations were found between performance on the Reading the Mind in the Eyes test (RMET) in participants with right hemisphere stroke.	30 (14:16); 67.76 (±12.24)	ICH 2, IS 28; R 15, L 15	N/A	N/A; Early subacute (74.24 ± 32.49)	ToM	The Reading the Mind in the Eyes Test	N/A	RHL 10.07 (±4.11) LHL 15.27 (±2.99)
						ToM	Eye Control Test		RHL 13.33 (±3.09) LHL 14.47 (±3.07)
Nijsse [53]; Inception cohort	Participants with stroke performed significantly worse on the ToM compared to controls. No significant differences were found between right and left hemisphere stroke patients on ToM tests.	148 (103:45); 63.9 (±11.3)	IS 137, ICH 11; R 62, L 50	Hemispheres 112 Vertebrobasilar 36	2.0 (±3.0), NIHSS; Chronic (range 1095–1462)	ToM	The Cartoon test		21.28 (±6.8)
						ToM	The Faux Pas test, Detection		9.25 (±1.0)
Nijsse [54], Cohort	Participants performed significantly worse on the ToM compared to controls. No significant differences were found between subgroups (vertebrobasilar vs. anterior circulation, left vs. right hemisphere). Significant but weak correlations were found between social cognition test results and DEX-proxy rating.	119 (85:34); 64.3 (±11.0)	IS 111, ICH 8; R 50, L 38	Hemispheres 88 Vertebrobasilar 31	2.0 (±3.1); Chronic (range 1095–1462)	ToM	The Cartoon test		21.04 (±6.9)
						ToM	The Faux Pas test, detection		9.21 (±1.0)
Sensenbrenner [60]; Observational	Impairment in ToM was common, 34.2 % of participants with stroke had difficulty with ToM.	43 (26:23); 66.8 (±15.1)	IS 38, ICH 5; R 13, L 16	Cerebral hemisphere 29, Brainstem or cerebellum 14	N/A; Chronic (1168 ± 73.05)	ToM	The Faux Pas test, detection	14 (34.2)	
Stiekema [64]; Longitudinal	ToM was not associated with restrictions in participation three to four years after stroke.	118 (85:33); 67.4 (±11.1)	IS 109, ICH 9; R 54, L 38	Cerebral hemisphere 92, Vertebrobasilar 23	2.0 (±3.0), NIHSS; Chronic (range 1096–1461)	ToM	Cartoon test		21.4 (±6.6)
						ToM	The shortened version of the Faux Pas test		9.3 (±1.1)
Surian [65]; Cross-sectional	Both patients with right hemisphere damage and left hemisphere damage performed well on the ToM tasks when presented with visual aids. However, patients with right hemisphere damage had difficulty when the same tasks were presented only verbally.	32 (17:15); 65.44 (±14.68)	IS 32; R 16, L 16	Frontal 6, Parietal 6, Temporal 2, Tempoparieral 3, Frontoparietal 4, Frontotemporal 3, Internal capsula 6, Parietal-occipital 2	N/A; Early subacute (≤60)	ToM	RHD, Implicit Condition, False, correct/error RHD, Implicit Condition, true, correct/error	7/1 8/0	
							RHD, Explicit condition, False, correct/error RHD, Explicit condition, True, correct/error	7/1 7/1	
							LHD, Implicit Condition, False, Correct/error LHD, Implicit Condition, True, Correct/error	7/1 8/0	
							LHD, Explicit Condition, False, Correct/error LHD, Implicit Condition, True, Correct/error	8/0 7/1	
						ToM	RHD, Implicit Condition, False, correct/error RHD, Implicit Condition, true, correct/error	7/1 8/0	
							RHD, Explicit condition, False, correct/error RHD, Explicit condition, True, correct/error	7/1 771	
							LHD, Implicit Condition, False, Correct/error LHD, Implicit Condition, True, Correct/error	8/0 8/0	
							LHD, Explicit Condition, False, Correct/error LHD, Implicit Condition, True, Correct/error	8/0 7/1	
Tompkins [71]; Experimental	Participants with a right hemisphere stroke had more difficulty understanding texts that involved thinking about others’ thoughts and feelings. They were slower and less accurate compared to people without brain damage.	22 (13:9); 64.4 (±10.3)	IS 13, ICH 9; R 22	Cortical anterior 3, Cortical posterior 1, Cortical mixed 2, Subcortical 8, Cortical & subcortical 2, MCA 6	N/A; Chronic (1999.91 ± 1588.97)	ToM	The “mental causal inference” stimuli		6.81 (±0.51)
						Mental-State Inferences	Mental inference stimuli, comparison		6.48 (±0.75)
						Causal Inferences	Sentence recognition task		12.67 (±3.0)
Yeh [77]; Case-control	Participants with stroke were impaired in both verbal and non-verbal ToM as compared to a control group.	34 (15:17); 60.82 (±11.21)	N/A; R 20, L 14	Basal ganglia 12, Frontal 5, Thalamus 6, Pons 6, Parietal 2, Frontoparietal 1, Occipitoparietal 1, Subcortical 1	N/A; Chronic (range 553–622)	*ToM* Verbal cognitive	The Faux Pas task	N/A	Right: 7.15 (±2.06) Left: 6.57 (±3.76)
						Non-verbal cognitive	Task with ten pictures: five pictures testing cognitive ToM and five pictures testing affective ToM		Right: 2.13 (±1.73) Left: 3.93 (±2.49)
Buunk [31]; Observational	Participants with aneurysmal subarachnoid hemorrhage (aSAH) showed significant impairments in ToM compared to healthy controls.	88 (27:61); 53.3 (±9.8)	SAH 88; N/A	Aneurysm circulation anterior 69, Posterior 19	18 (20.5 %) WFNS score 4–5/high; Late subacute (143.07 ± 54.79)	ToM	The Cartoon Test	N/A	9.1 (±3.7)
						Inappropriate behavior: detection	A shortened version of the Faux Pas Test	N/A	8.7 (±1.4)
Tsolakopoulos [72]; Cros-sectional	Participants with stroke had clear impairments in the cognitive aspects of ToM, which were closely linked to difficulties in understanding indirect requests and metaphors. Affective ToM was less consistently affected. All patients with pragmatic deficits also had impairments in either ToM or executive functions. Overall, ToM impairments varied across individuals, highlighting the heterogeneity in cognitive profiles.	25 (15:10); 58.4 (±12.04)	N/A; R 25	Multiple loci 18, Inferior parietal lobule 1, Precentral gyrus 1, Internal capsule 1, N/A	N/A; Chronic (≥182)	ToM	The Frith–Happé animation test	16 (64 %)	N/A
						ToM Cognitive	The Greek brief version of the faux pas test		
					
Xi [76]; Case-control	Participants with the temporal lobe cerebral infarction (TLCI) group performed significantly worse than the control group in ToM tasks, particularly in understanding faux pas. There were no significant differences in control questions, identifying faux pas, or gender recognition.	19 (16:3); 55.16 (±14.04)	IS 19; R 15, L 4	Temporal lobe 19	4.68 (±2.94), NIHSS; Early subacute (36.42 ± 8.91)	ToM	The adapted Stone’s Faux Pas task	N/A	N/A
					
Pluta [58]; Cross-sectional	Participants with stroke had difficulties with all ToM tasks. Dysfunctions in pragmatic competence, and to a lesser extent executive functions, significantly contribute to ToM impairments.	58 (32:26); 54.50 (±14.84)	N/A; R 29, L 24, B 5	N/A	N/A; Chronic (range 395.72–864.50)	ToM, Mental questions	18 short vignettes designed based on available tasks in the literature and original tasks that authors developed.	N/A	61.36 (±14.68)
						ToM, Cognitive components			27.12 (±7.12)
	
						ToM, Control questions			32.89 (±3.18)
Balaban [23]; Cross-sectional	Among participants with right hemisphere stroke, 68 % had ToM impairments. Despite the ToM impairments, all patients showed intact syntactic abilities, particularly in sentence embedding.	25 (17:8); 53 (±11)	IS 20, ICH 1, Both 3, Other 2; R 25	N/A	N/A; Early subacute (≥60)	ToM, False belief ( % correct)	The aToMia battery	N/A	84 (±31)
						Second-order false belief			64 (±41)
						Knowledge gaps			56 (±51)
						Faux pas			48 (±47)
						Surprise			70 (±43)
						Mental state cartoon			36 (±47)
Humphreys [43]; Experimental	Participants with impaired ToM showed differences in their ability to process social cues and coordinate actions with others. Those with posterior parietal cortex lesions struggle to maintain attention to social cues, while those with frontal region lesions have difficulty sustaining the coding of another’s actions over time	18 (16:2); 63.94 (±13.29)	IS/ICH 15, Anoxia 2, Other 2; R 17, L 1	Frontal 6, Posterior parietal cortex/temporo-parietal junction 6, Unclear 6	N/A; N/A	ToM	Experimental, no specific instrument	N/A	N/A
Baldo [24]; Cross-sectional	Responses of participants with RHD were rated as significantly less appropriate than controls and were also significantly less typical than controls and participants with LHD. Participants with RHD produced a numerically lower proportion of formulaic expressions than controls, but this difference was only a trend.	22 (18:4); 67.05 (±13.6)	N/A; R 11, L 11	N/A	N/A: Chronic (365)	Interpretation of social situations	Cartoon test: 12 large, black-and-white, hand drawn.	N/A	N/A
Weed [75] Experimental	People with stroke displayed a reduced ability to discriminate between the stimulus categories, and a bias toward reduced ascription of mental states in the ToM condition.	11 (8:3); 60.64 (±7.68)	ICF 4, IS 5, Both 2; R 11	Frontal 4, Temporo-parietal 1, Fronto-parieto-temporal 1, Other 5	N/A; Early to late subacute (range 30–210)	ToM	Eight animations: the four ‘Random’ animations and the four ‘ToM’ animations.	N/A	N/A

**Abbreviations:** M, male; F, female; SD, standard deviation; IQR, interquartile range; n, number; R, right; L, left; B, bilateral; N/A, data not available or could not be extracted; IS, ischemic stroke; ICH, intracerebral hemorrhage; SAH, subarachnoid hemorrhage; aSAH, aneurysmal subarachnoid hemorrhage; NA = not applicable / Not specified; RHD, right hemisphere damage; LHD, left hemisphere damage; MCA, middle cerebral artery; PCA, posterior cerebral artery; PICA, posterior inferior cerebellar artery; NIHSS, National Institutes of Health Stroke Scale; WFNS, World Federation of Neurological Surgeons grading scale; ToM, Theory of Mind; RMET, Reading the Mind in the Eyes Test; AHP, anosognosia for hemiplegia; HP, hemiplegia; DEX, Dysexecutive Questionnaire;.

#### 3.2.3 Empathy

Empathy was explored in 15 studies [21,23,45,47,50,53,54,[56], [57], [58], [59],^64^,^72^,^76^,^77^], and most (*n* = 23, 60 %) were conducted during the chronic phase of stroke (Table 4). Commonly applied assessment instruments were the Interpersonal Reactivity Index [47,50,57,77], Balanced Emotional Empathy Scale [53,54,64], and Faux Pas Test (description of feelings) [53,54,64,72,77]. The prevalence of impairment ranged 6–58 %. However, as Table 4 shows, some studies revealed impaired empathy in stroke survivors [45,47,50,[56], [57], [58], [59],^72^,^77^], unlike others that found no such impairment [21,23,53,54,76].

**Table 4:** Characteristics of studies on empathy in stroke survivors.

Author (year); design	Major results related to social cognitive function	No. of patients (M: F); age mean (±SD)/median (IQR), years	Type of stroke; lateralization, n	Stroke location, n	Stoke severity, instrument name; and phase (days)	Social cognitive function domain	Name of the instrument	Prevalence of impairment, n ( %)	Unit of measurement (median/IQR, mean/±SD)
Adams [21]; Case-Control	Among participants with stroke, self-reported capacity for affective empathy was intact.	35 (23:12); 64.69 (±12.92)	ICH 5, IS 30; R 18, L 17	Frontal lobe 5, Parietal lobe 3, Occipital lobe 3, Cerebellum 6, Basal ganglia 1, Multi-lobe 8, Subcortical/other structures 7, N/A 2	N/A; Chronic (415.20 ± 415.20)	Affective empathy	The Empathic Concern subscale of the Interpersonal Reactivity Index.	N/A	21.06 (±3.94)
Kliszcz [42]; Experimental	Emotional empathy was positively related with asymmetry index performance, but there was no significant link between cognitive empathy and asymmetry index.	21 (18:3); 55.8 (±7.60)	IS 21; R 21	N/A	N/A; Early subacute (N/A)	Emotional empathy (EM): sympathetic tendency	33 items questionnaire measure of emotional empathy	N/A	6.5 (±4.01)
						EM: Appreciation of the feelings of unfamiliar and distant others			8.1 (±5.39)
						EM: Extreme emotional responsiveness			8.9 (±4.85)
						EM: Willingness to be in contact with other who have problems			8.95 (±4.56)
						EM: Tendency to be moved by others` positive emotional experiences			10.75 (±6.15)
						EM: Susceptibility to emotional contagion			11.15 (±4.68)
						EM: Tendency to be moved by others` negative emotional experiences			13.8 (±5.14)
						Cognitive empathy (CE): Fantasy scale	Interpersonal reactivity index		29.5 (±3.66)
						CE: Personal distress			22.45 (±7.35)
						CE: Perspective taking			30.75 (±4.25)
						CE: Empathic concern			27.65 (±3.81)
Nijsse [53]; Inception cohort	Participants with stroke did not show significant deficits in empathy compared to controls.	148 (103:45); 63.9 (±11.3)	IS 137, ICH 11; R 62, L 50	Hemispheres 112, Vertebrobasilar 36	2.0 (±3.0), NIHSS; Chronic (range 1095–1462)	Empathy	The Faux Pas test	N/A	3.00 (±1.2)
						Emotional empathy	The Dutch version of the Balanced Emotional Empathy Scale		32.06 (±22.5)
Nijsse [54], Cohort	There were no significant differences between stroke patients and controls regarding empathy. However, proxy reports indicated more behavioral problems related to empathy, with lower empathy scores correlating with greater observed difficulties	119 (85:34); 64.3 (±11.0)	IS 111, ICH 8; R 50, L 38	Hemispheres 88, Vertebrobasilar 31	2.0 (±3.1); Chronic (range 1095–1462)	Empathy	The Faux Pas test	N/A	2.99 (±1.2)
						Emotional empathy	The Dutch version of the Balanced Emotional Empathy Scale		3.02 (±1.8)
Pertz [57]; Case-control	Patients with stroke reported overall higher degrees of alexithymia and more personal distress in response to other individuals’ emotional suffering as assessed by self-report.	36 (24:12); 57.9 (±8.5)	IS 30, ICH 6; R 13, L 19, B 4	N/A	N/A; Chronic (730 range 120–2920)	Interpersonal Reactivity (IR): Empathic concern	The Interpersonal Reactivity Index (German version)	2 (5.7)	14.6 (±2.1)
						IR: Personal distress		3 (8.6)	11.8 (±2.5)
						IR: Fantasy		12 (34.3)	11.5 (±2.6)
						IR: Perspective taking		4 (11.4)	14.2 (±2.9)
Qu [59]; Observational	A smaller putamen volume was associated with a more severe impairment of empathy.	41 (23:18); 61.73 (± 9.02)	IS 41; R 36, L 44	Cortical 18, Subcortical 31, Infratentorial 8, Frontal lobe 10, Temporal lobe 8, Parietal lobe 9, Occipital lobe 4.	2 (0–3), NIHSS; Early subacute (≤90)	Empathy	The Chinese version of the Empathy Quotient	N/A	N/A
Stiekema [64]; Longitudinal	Empathy-related abilities were not significant independent predictors of participation 3–4 years post-stroke.	118 (85:33); 67.4 (±11.1)	IS 109, ICH 9; R 54, L 38	Cerebral hemisphere 92, Vertebrobasilar 23	2.0 (±3.0), NIHSS; Chronic (range 1096–1461)	Empathy	The Faux Pas test (short version)	N/A	2.9 (±1.2)
							The Balanced Emotional Empathy Scale		32.4 (±22.0)
Yeh [77]; Case-control	Right hemisphere stroke was associated with significant deficits in cognitive empathy, particularly in perspective-taking. Affective empathy was relatively unaffected by stroke.	34 (15:17); 60.82 (±11.21)	N/A; R 20, L 14	Basal ganglia 12, Frontal 5, Thalamus 6, Pons 6, Parietal 2, Frontoparietal 1, Occipitoparietal 1, Subcortical 1	N/A; Chronic (range 553–622)	*Empathy*: Cognitive	The Interpersonal Reactivity Index	N/A	Right: 5.36 (±1.01) Left: 5.83 (±1.66)
						Perspective-taking			Right: 3.09 (±1.01) Left: 3.52 (±1.04)
						Fantasy			Right: 2.27 (±1.25) Left: 2.3 (±0.839
						Emotional empathy			Right: 6.27 (±1.38) Left: 5.60 (±1.60)
						Personal distress			Right: 3.11 (±1.28) Left: 2.64 (±1.15)
						Empathic concern			Right: 3.16 (±0.75) Left: 2.95 (±1.03)
						Verbal affective (ToM)	The Faux Pas task		Right: 6.90 (±2.77) Left: 6.29 (±3.63)
						Non-verbal affective (ToM)	Task with ten pictures: five pictures testing cognitive ToM and five pictures testing affective ToM		Right: 4.00 (±1.02) Left: 3.73 (±1.87)
Tsolakopoulos [72]; Cros-sectional	Cognitive empathy was significantly impaired in RBD patients.	25 (15:10); 58.4 (±12.04)	N/A; R 25	Multiple loci 18, Inferior parietal lobe 1, Precentral gyrus 1, Internal capsule 1, N/A 4	N/A; Chronic (≥182)	Cognitive empathy	The test with 5 faux pas stories and 5 control stories	N/A	N/A
						Affective ToM	The Faux Pas test (the Greek brief version)	N/A	N/A
Leigh [50]; Case-control	Affective empathy impairment was linked to infarcts in the temporal pole and anterior insula. All patients with this impairment also struggled with affective prosody comprehension, though some with prosodic issues had intact empathy.	27 (18:9); 54.5 (±15.5)	IS 27; R 27	Prefrontal cortex 3, Anterior cingulate 2, Amygdala 4, N/A 18	N/A; Acute (≤2)	Affective empathy	The Affective Empathy Task	14 (58.33)	N/A
						Cognitive empathy	The Interpersonal Reactivity Index	N/A	
Balaban [23]; Cross-sectional	Overall empathy scores were not significantly impaired, however a subset of patients showed clear deficits in empathic reasoning.	25 (17:8); 53 (±11)	IS 20, ICH 1, Both 3, Other 2; R 25	N/A	N/A; Early subacute (≥60)	Empathy	1st order		82 (±24)
							2nd order		64 (±47)
Xi [76]; Case-control	There were no significant differences in control questions, identifying faux pas, or gender recognition. These findings suggest that patients with TLCI struggle with interpreting others’ mental states based on eye expressions.	19 (16:3); 55.16 (±14.04)	IS 19; R 15 L 4	Temporal lobe 19	4.68 (±2.94), NIHSS; early subacute (36.42 ± 8.91)	Affective ToM	The Chinese version of the Reading the Mind in Eyes Task	N/A	N/A
Jospe [45]; Experimental	Patients with left-hemisphere damage have long-lasting difficulties comprehending real-world complex emotional situations.	24 (17:7); 65.13 (±8.76)	IS 14, ICH 8, Other 2; R 13, L 11	N/A	N/A; Chronic (1160.68 ± 714.73)	Understand the emotions	Empathic accuracy task	N/A	N/A
Pluta [58]; Cross-sectional	Stroke patients struggled with affective components of ToM.	58 (32:26); 54.50 (±14.84)	N/A; R 29 L 24 B 5	N/A	N/A; Chronic (range 395.72–864.50)	Affective components, ToM	18 short vignettes designed based on available tasks in the literature and original tasks that authors developed		34.24 (±8.83)
Oishi [56]; Cohort	Damage to certain white matter tracts, along with age and education, influenced the error rate in emotional empathy tasks. Damage to the uncinate fasciculus was the only factor independently associated with higher error rates.	30 (N/A); N/A	N/A; R 30	N/A	N/A; Acute (1)	Affective perspective taking	Participants were asked yes/no and multiple-choice questions requiring inferences about emotions of individuals in short videotapes or stories that were read to them.	19 (64)	N/A

Abbreviations: M, male; F, female; SD, standard deviation; IQR, interquartile range; n, number; R, right; L, left; B, bilateral; N/A, data could not be extracted / not available; IS, ischemic stroke; ICH, intracerebral hemorrhage; SAH, subarachnoid hemorrhage; RHD, right hemisphere damage; LHD, left hemisphere damage; NIHSS = National Institutes of Health Stroke Scale; WFNS, World Federation of Neurological Surgeons grading scale; ToM, Theory of Mind; IR, Interpersonal Reactivity (or Reactivity Index); TLCI, temporal lobe cerebral infarction.

#### 3.2.4 Emotion regulation

Table 5 shows that emotion regulation was examined in one study [35]. Cooper et al. [35] applied the Difficulties in Emotion Regulation Scale during early subacute phase. The reported mean emotion regulation difficulty scores ranged between 8.85 (s.d. ±3.87) and 72.07 (s.d. ±21.54).

**Table 5:** Characteristics of the study on emotion regulation in stroke survivors.

Author (year); design	Major results related to social cognitive function	No. of patients (M:F); age mean (±SD)/median (IQR), years	Type of stroke; lateralization, n	Stroke location, n	Stoke severity, instrument name; and phase (days)	Social cognitive function domain	Name of the instrument	Prevalence of impairment, n ( %)	Unit of measurement (median/IQR, mean/±SD)
Cooper [35]; Mixed methods	In the acute stage of stroke, participants had significant emotion regulation difficulties linked to social participation issues. In the chronic stage, these difficulties continued to affect social participation, but no single aspect of emotion regulation predicted long-term social participation.	Aim I: 75 (47:28); 66.22 (±12.13)	N/A; N/A	N/A	I: N/A; Early subacute (63 (±36)	I: Emotion regulation	The Difficulties in Emotion Regulation Scale	N/A	72.07 (±21.54)
		Aim II: 48 (28:20); 67.63 (±12.92)	N/A; N/A	N/A	II: N/A; Chronic 518 (±91)	II: Non-acceptance	The Difficulties in Emotion Regulation Scale	N/A	12.50 (±6.70)
						Goals			9.75 (±3.74)
						Impulse			8.59 (±3.14)
						Awareness			15.74 (±4.54)
						Strategy			13.04 (±4.78)
						Clarity			8.85 (±3.87)

**Abbreviations:** M, male; F, female; SD, standard deviation; IQR, interquartile range; *n*, number; R, right; L, left; B, bilateral; N/A, data could not be extracted; RHD, right hemisphere damage; LHD, left hemisphere damage; IS, ischemic stroke; ICH, intracerebral hemorrhage; SAH, subarachnoid hemorrhage; NIHSS, National Institutes of Health Stroke Scale; WFNS, World Federation of Neurological Surgeons (grading scale).

#### 3.2.5 Social problem-solving and interaction

As Table 6 shows, this function was documented in two studies that assessed social problem-solving fluency, the detection and subjective evaluation of social awkwardness, selection of optimal social responses, and interpretation of social situations [57,63]. The assessment instruments, the Social Problem-Solving Fluency Task (abbreviated German version) [57] and Functional Independence Measure (FIM) [63] used to assess social cognitive domain, were administered during the chronic phase of stroke. As reported, stroke survivors had difficulty generating high-quality social solutions and detecting socially awkward situations, despite their understanding of tasks and being able to select appropriate responses [57]. The mean scores of the impairment ranged from 18.02 (s.d. ±4.62) to 98.1 (s.d. ±4.0).

**Table 6:** Characteristics of studies on social problem-solving and interaction in stroke survivors.

Author (year); design	Major results related to social cognitive function	No. of patients (M:F); age mean (±SD)/median (IQR), y	Type of stroke; lateralization, n	Stroke location, n	Stoke severity, instrument name; and phase (days)	Social cognitive function domain	Name of the instrument	Prevalence of impairment, n ( %)	Unit of measurement (median/IQR, mean/±SD)
Pertz ([57] Case-control	Participants with stroke had difficulty generating high-quality social solutions, particularly social-related problems, and detecting socially awkward situations, despite understanding the tasks and selecting appropriate responses. These issues persisted even when controlling for intelligence and verbal fluency, with elevated alexithymia possibly playing a role.	36 (24:12); 57.9 (±8.5)	IS 30, ICH 6; R 13, L 19, B 4	N/A	N/A; Chronic (730 range 120–2920)	Social problem solving (SPS): Control questions	The Social Problem-Solving Fluency Task (abbreviated German version)	N/A	98.1 (±4.0)
						SPS: Detection of awkwardness			79.4 (±24.6)
						SPS: Subjective degree of awkwardness			66.2 (±14.9)
						SPS: Selection of optimal alternatives			56.1 (±21.3)
Souza [63]; Cohort	Impairment in social cognition can predict the restriction of post-stroke social participation in the community.	48 (27:21); 65 (58–78)	IS 43, ICH 5; N/A	N/A	4 (1–7), NIHSS; Chronic (365)	Social Cognition	Social cognition, Functional Independence Measure Domaine	N/A	18.02 (±4.62)

**Abbreviations:**M, male; F, female; SD, standard deviation; IQR, interquartile range; *n*, number; R, right; L, left; B, bilateral; N/A, data could not be extracted; B, both; RHD, right hemisphere damage; LHD, left hemisphere damage; SP, social problem.

## 4 Discussion

The aim of this scoping review was to provide a comprehensive overview of the current knowledge on social cognitive function in stroke survivors. We included 62 studies; the study samples comprised survivors of ischemic stroke, hemorrhagic stroke, and/or subarachnoid hemorrhage. Five domains of social cognitive functions were identified, with emotion perception and recognition being the most frequently studied category. The Ekman 60-Faces Test was the most applied assessment tool. The findings showed that impairments in social cognitive function were common sequalae after stroke (7–100 %) across all types of stroke, regardless of the lesion location or time elapsed post index stroke. Furthermore, no studies investigated rehabilitation intervention techniques to address these deficits.

Across the 62 original studies included in this scoping review, there was evidence of a somewhat selective patient population rather than a representative broader population of those with stroke. Most studies imposed inclusion and exclusion criteria, which excluded individuals with prior neurological or psychiatric disorders, severe stroke, aphasia, cognitive impairments, or sensory deficits—which inherently could have biased the samples toward higher-functioning individuals. Many also required first-ever, unilateral strokes (often in the right hemisphere), and fluency in the study language to ensure reliable neuropsychological assessment. While these methodological controls enhance internal validity, they limit external validity and may underestimate the true prevalence and diversity of social cognitive deficits after stroke. Consequently, the findings primarily reflect a subset of stroke survivors with relatively preserved cognitive and communicative abilities, suggesting that the full impact of stroke on social cognition in more impaired or diverse populations remains underexplored.

To facilitate clarity, we based the results of this scoping review on five major domains of social cognitive function: emotion perception and recognition, ToM, empathy, emotion regulation, and (5) social problem-solving and interaction. Among these, the most frequently studied domain was emotion perception and recognition, while social problem-solving and interaction were the least studied. Our categorization along these domain groups partially aligns with that of Wallis et al. [79], who identified the following social cognitive function domains: emotion perception, ToM, social communication, identity recognition, and empathy. This categorization of specific social cognitive function domains could be argued; for instance, it is debatable whether affective ToM should be considered a subdomain of ToM or empathy. However, our categorization was derived from the available data and selected for clarity and ease of presentation. As Pinkham et al. [1] suggested, social cognitive functions are better distinguished by their level of information processing than by specific domains. These processes entail both "hot" emotional components and "cold" cognitive reasoning, supported by underlying neural networks that are both distinct and overlapping [1]. Overall, these conceptual ambiguities highlight the urgent need for a comprehensive and standardized conceptual analysis of social cognitive functions in individuals with stroke.

Clinical and cohort characteristics could be major sources of the wide prevalence ranges observed in this scoping review. Across studies, lesion location revealed an association with deficits in social cognition, including that the right-hemisphere/fronto-temporo-insular damage and temporoparietal regions were repeatedly implicated in impaired facial emotion recognition, prosody, and ToM. These findings are consistent with voxel-based lesion-symptom and anatomical models. Meta analysis by Adams et al. [11] showed moderate–large stroke-related impairments in ToM (*r*≈−0.44), social perception (*r*≈−0.55), and social behavior (*r*≈−0.53), with affective empathy not reliably impaired. These effects were robust across left vs. right lateralized lesions. Converging lesion-mapping and systematic reviews further implicate the fronto-temporo-insular and temporoparietal regions—especially in the right hemisphere—for emotion and affective prosody processing. These findings reinforce the critical roles of these cortical hubs and their link with a higher observed prevalence of these deficits in cohorts enriched for lesions in such areas [11]. Across the included studies, acute/early subacute stroke cohorts had higher impairment prevalence relative to later-phase cohorts, but persistent deficits were still detectable years after stroke in many samples. This pattern aligns with the Stroke Recovery and Rehabilitation Roundtable framework [16], which defines biologically informed recovery windows and highlights the early weeks as a period of rapid, largely spontaneous improvement followed by slower gains. This implies that point-prevalence estimates could be stroke recovery phase-dependent [16].

In this scoping review, we identified a broad range of instruments and experimental tests for assessing various domains of social cognitive functioning. The tools we described, along with their frequency of use, align with the findings of Wallis et al. [79] who conducted a scoping review on social cognition following acquired brain injury. The most frequently used performance test was the Ekman 60-Faces. This test relies on static, posed photographs developed in the 1970s. Its outdated stimulus set, and restricted cultural range may limit ecological validity and be confusing to contemporary, diverse patient populations. More broadly, social-cognition measures are highly sensitive to cultural and linguistic context (stimuli, translations, and normative data), and in clinical practice, clinicians often supplement test performance with structured observation and collateral interviews to capture real-world impact. The only stroke rehabilitation-specific instrument that we identified was the FIM [63]. The FIM has a cognitive domain, referred to as “social cognition.” This social cognition domain comprises social interaction, which involves “getting along and interacting with others in social or therapeutic situations“; problem solving, which refers to “solving problems and making responsible decisions associated with day-to-day activities”; and “memory.” However, it is debatable whether these domain items adequately capture social cognitive processes, as the FIM scale primarily assesses functional performance. Nevertheless, we retained the FIM in our review because one of the included studies [63] explicitly used the social interaction item as a proxy for social cognition. Its inclusion should therefore be understood as reflecting current practice rather than an evidence of construct validity. It also highlights the need for more targeted instruments in this context.

We observed considerable variability in assessment instruments and definitions across studies. The same social‐cognition domains were operationalized using instruments that differed in stimuli, task demands, and scoring rules. This heterogeneity resulted in non-comparable scores and classification rates across studies. Accordingly, the prevalence should be interpreted conditioned on both the clinical characteristics and specific instrument used for assessing each social cognitive domain. These measurement differences imply that impairment designations can depend more on the choice of test than on the actual patient status. Moreover, such heterogeneity biases pooled prevalence estimates and obscures true moderators of impairment. To improve comparability cross studies, adopting core outcome sets, stratifying analyses by the type of instrument and recovery phase, and conducting threshold-sensitivity analyses are recommended approaches. This heterogeneity is an important finding of our study, as it highlights the current lack of consensus in the field and underscores the need for standardized definitions and assessment approaches for social cognitive function in patients with stroke. Establishing such standards is crucial for developing effective rehabilitation programs.

Our definition of social cognitive functioning is similar to that of studies from various disciplines and patient groups, including those with mild cognitive impairment and dementia [80] and neurodevelopmental disorders [81], as well as the fields of psychiatry [82,83] and neurology [12]. In line with our scoping review, these previous studies reported varying degrees of impairments in social cognitive function across domains [12,80,82,83]. This consistency in findings across different patient groups and research disciplines suggests that social cognitive deficits may arise from fundamental neural mechanisms. This idea can be understood through the biopsychosocial paradigm, as described by Deen et al. [84], suggesting that the primate brain contains specialized and hierarchical networks dedicated to social cognition, ranging from individual neurons to interconnected brain regions, collectively known as the social brain [84]. They emphasized that the anatomical organization of the social brain closely resembles overlapping nonsocial systems, suggesting that similar computational processes are at work in both domains. Accordingly, we can infer that any type of stroke, regardless of the specific brain region affected, could plausibly disrupt these shared computational mechanisms, which can in turn, lead to impairments in social cognitive function.

There are several strengths and limitations of this scoping review. We followed established guidelines in the planning and conducting of the review [13,85]. The search terms were conceptualized and tested in collaboration with a librarian, and the search was conducted across multiple major databases (e.g., MEDLINE, PsycINFO, EMBASE). MeSH terms and relevant synonyms were used to capture the full scope of relevant studies from year 2000 onward. The entire review processes, from title and abstract screening to data extraction, were performed independently by two authors, to ensure reliability of the results. Moreover, a risk of bias assessment, which is usually not required in a scoping review, was not conducted [14,86]. Nevertheless, certain limitations should be acknowledged. One is the potential for selection bias, as only studies published in English were included.

In conclusion, we identified several critical areas requiring further research on social cognitive function following stroke. There is a clear need for a comprehensive, conceptually grounded analysis of how the term social cognition should be defined and applied in stroke-related research and clinical practice. Such conceptually grounded framework could help establish a common vocabulary for discussing social cognition in stroke care services. Moreover, this could also lead to the development of stroke-specific assessment instruments. Additionally, the lack of standardized, stroke-specific assessment tools underscores the importance of developing and validating reliable measures. Stroke affects the brain area and network crucial for social cognition [6,7], resulting in diverse impairments that may differ from those observed in other neurological or psychiatric conditions [80,82,83]. However, many stroke survivors also experience broader cognitive and mood-related impairments, which may complicate the accurate identification and assessment of social cognitive deficits. Therefore, stroke-specific tools are essential to clearly distinguish social cognitive deficits from broader cognitive and emotional disturbances. Developing these targeted measures could facilitate precise diagnosis and the identification of a precondition for targeting when developing rehabilitation programs. Longitudinal studies involving large cohorts covering both acute and chronic phases are also essential to deepen our understanding of changes overtime in social cognitive function and how impairments affect body functions, daily activities, and overall quality of life after stroke. Furthermore, certain domains, particularly social interaction and social problem-solving, remain significantly underexplored, despite their critical role in functional recovery and successful community reintegration. The lack of intervention studies underscores the need to develop and test clinic-ready programs for post-stroke social cognition. Future work should therefore progress beyond mapping deficits and examine targeted approaches, including structured rehabilitation programs addressing the Theory of Mind or emotion recognition. Group-based and peer-supported modules may increase ecological validity, whereas embedding social cognition training within established neurorehabilitation protocols could facilitate implementation in routine care.

In summary, beyond study-level limitations, our findings highlight broader methodological challenges in the field of social cognition assessment. Social cognition remains a developing cognitive domain, with ongoing debates about construct definitions, diversity of instruments, and limited standardization of administration and scoring procedures. These factors contribute to inconsistencies across studies and complex comparisons. Therefore, the variability reported in this review should be interpreted not only as a limitation of the included evidence but also as a reflection of the evolving state of the field, underscoring the need for conceptual clarity and psychometric refinement in future research.

## Funding

This work was supported by the Swedish State under an agreement between the Swedish Government and the County Councils, the ALF agreement (ALFGBG-877961, ALFGBG-1006984). The sponsors had no role in the design and conduct of the study, the collection, management, analysis, and interpretation of the data.

## Availability of data, code, and other materials

This scoping review did not generate new data, specific codes, or other materials. Full texts of included studies, data extraction files, and other related materials can be requested form the corresponding author (tamar.abzhandadze@gu.se).

## Ethical standard

The manuscript does not contain clinical studies or patient data.

## Consent to participate

Not applicable.

## CRediT authorship contribution statement

**Ana Davlasheridze:** Writing – review & editing, Writing – original draft, Formal analysis, Data curation. **Lena Rafsten:** Writing – review & editing, Methodology, Formal analysis, Data curation. **David Krabbe:** Writing – review & editing, Formal analysis, Data curation. **Farzaneh Badinlou:** Writing – review & editing, Formal analysis, Data curation. **Renate Reniers:** Writing – review & editing, Methodology, Conceptualization. **Terence J Quinn:** Writing – review & editing, Supervision, Methodology, Formal analysis, Conceptualization. **Tamar Abzhandadze:** Writing – review & editing, Writing – original draft, Visualization, Supervision, Project administration, Methodology, Investigation, Funding acquisition, Formal analysis, Data curation, Conceptualization.

## Declaration of competing interest

The authors declare that they have no conflicts of interest.

## Appendix Supplementary materials

Image, application 1
