# Supplementary material for: Social cognitive function in stroke survivors: A scoping review
Source: Cereb Circ Cogn Behav. 2025 Sep 22;9:100398. doi: 10.1016/j.cccb.2025.100398 (PMC12514548; doi:10.1016/j.cccb.2025.100398)
Supplement: Supplementary file 1 [file mmc1.docx]

# Supplementary material

Supplementary Table S1. Information sources and dates of the search

Electronic bibliographic databases and clinical trial registers searched during (26/02/2024) included (presented in alphabetical order):

Cochrane library: the Cochrane Central Register of Controlled Trials (CENTRAL), and the Cochrane Database of Systematic Reviews (CDSR) in the Cochrane Library (the Cochrane Library Issue 2 of 12, February 2024)

EMBASE (Ovid, from 1/1/2000 to 23/02/2024)

MEDLINE (Ovid, from 1/1/2000 to 26/02/2024)

CINAHL (EBSCO, from 1/1/2000 to 26/02/2024)

AMED (EBSCO, from 1/1/2000 to 26/02/2024)

PsycINFO (EBSCO, from 1/1/2000, Publication Type: All Journals; Publication Year: 2000–2024)

Web of Science (Core Collection, from 1/1/2000 to 26/02/2024).

Supplementary Table S2. Full electronic search strategy and search terms

The literature search was conducted by a librarian at the Medical Library of Sahlgrenska University Hospital, Sweden. The keywords/search terms were formulated by the last author in consultation with the librarian. Additionally, the librarian conducted an initial literature search to refine and enhance the accuracy of the keywords/search terms.

**2.1 Database:** Ovid MEDLINE(R) ALL (OvidSP)

**Date:** 2024-02-26

**No of results:** 6464

| **#** | **Searches** | **Results** |
| --- | --- | --- |
| 1 | cerebrovascular disorders/ or exp basal ganglia cerebrovascular disease/ or exp brain ischemia/ or exp carotid artery diseases/ or exp cerebral small vessel diseases/ or exp intracranial arterial diseases/ or exp "intracranial embolism and thrombosis"/ or exp intracranial hemorrhages/ or stroke/ or exp brain infarction/ or exp stroke, lacunar/ or exp vasospasm, intracranial/ or exp vertebral artery dissection/ | 422908 |
| 2 | (stroke$ or poststroke or apoplex$ or cerebral vasc$ or brain vasc$ or cerebrovasc$).ab,kf,ti. | 397125 |
| 3 | ((brain$ or cerebr$ or cerebell$ or vertebrobasil$ or hemispher$ or intracran$ or intra-cran$ or intracerebral or infratentorial or infra-tentorial or supratentorial or supra-tentorial or middle cerebral arter$ or MCA$ or anterior circulation$ or posterior circulation$ or basilar arter$ or vertebral arter$ or space-occupying or spaceoccupying) adj3 (isch?emi$ or infarct$ or thrombo$ or emboli$ or occlus$ or hypoxi$)).ab,kf,ti. | 128797 |
| 4 | ((brain$ or cerebr$ or cerebell$ or intracerebral or intracran$ or intra-cran$ or parenchymal or intraparenchymal or intraventricular or intra-ventricular or infratentorial or infra-tentorial or supratentorial or supra-tentorial or basal gangli$ or putaminal or putamen or posterior fossa or hemispher$ or subarachnoid) adj3 (h?emorrhag$ or h?ematoma$ or bleed$)).ab,kf,ti. | 95770 |
| 5 | hemiplegia/ or exp paresis/ or exp Gait Disorders, Neurologic/ | 28871 |
| 6 | (hemipleg$ or hemipar$ or paresis or paraparesis or paretic).ab,kf,ti. | 48684 |
| 7 | 1 or 2 or 3 or 4 or 5 or 6 | 712447 |
| 8 | social cognition/ or exp social perception/ or exp facial expression/ or exp facial recognition/ or exp social behavior/ or exp theory of mind/ or exp mentalization/ or exp empathy/ | 353161 |
| 9 | (social adj3 cogn$).ab,kf,ti. | 27904 |
| 10 | (social adj3 percep$).ab,kf,ti. | 7126 |
| 11 | (emotion$ adj3 percep$).ab,kf,ti. | 5230 |
| 12 | (emotion$ adj3 recogn$).ab,kf,ti. | 7629 |
| 13 | ((facial$ or face) adj3 expression$).ab,kf,ti. | 12763 |
| 14 | ((facial$ or face) adj3 recogn$).ab,kf,ti. | 9491 |
| 15 | (social$ adj3 behav$).ab,kf,ti. | 42950 |
| 16 | (prosod$ or speech$).ab,kf,ti. | 104625 |
| 17 | (theor$ adj3 (mind or minds)).ab,kf,ti. | 6564 |
| 18 | (mentali#ing$ or mentali#ation$ or (ment$ adj3 process$)).ab,kf,ti. | 8596 |
| 19 | (pragmat$ adj3 impairment$).ab,kf,ti. | 220 |
| 20 | ((nonliteral$ or literal$) adj3 language$).ab,kf,ti. | 177 |
| 21 | (sarcasm$ or lie$ or joke$).ab,kf,ti. | 99741 |
| 22 | empath$.ab,kf,ti. | 23816 |
| 23 | (perspect$ adj1 tak$).ab,kf,ti. | 2924 |
| 24 | 8 or 9 or 10 or 11 or 12 or 13 or 14 or 15 or 16 or 17 or 18 or 19 or 20 or 21 or 22 or 23 | 633178 |
| 25 | 7 and 24 | 9729 |
| 26 | animals/ not (animals/ and humans/) | 5164410 |
| 27 | 25 not 26 | 9508 |
| 28 | child/ not (child/ and adult/) | 1268260 |
| 29 | 27 not 28 | 9107 |
| 30 | (comment or editorial or letter).pt. | 2228754 |
| 31 | 29 not 30 | 8964 |
| **32** | **limit 31 to (yr="2000–Current" and (English or Swedish))** | **6464** |
| **exp/** = term from the Medline controlled vocabulary, including terms found below this term in the MeSH hierarchy.  **/** = term from the Medline controlled vocabulary, does not include terms found below this term in the MeSH hierarchy  **adj1 / adj3** = next to each other, in any order, up to 0 / 2 word(s) in between  **.ab,kf,ti**. = abstract, author keyword and title  **$** = truncation of word for alternate endings  **#** = retrieves all possible variations of a word in which the wildcard is present in the specified place. | | |

**2.2 Database:** The Cochrane Library Issue 2 of 12, February 2024
**Date:** 2024-02-26
**No of results:** 858, *Cochrane reviews: 34 and Trials: 824.*

| **ID** | **Search** | **Hits** |
| --- | --- | --- |
| #1 | MeSH descriptor: [Cerebrovascular Disorders] this term only | 1851 |
| #2 | MeSH descriptor: [Basal Ganglia Cerebrovascular Disease] explode all trees | 37 |
| #3 | MeSH descriptor: [Brain Ischemia] explode all trees | 5814 |
| #4 | MeSH descriptor: [Carotid Artery Diseases] explode all trees | 1760 |
| #5 | MeSH descriptor: [Cerebral Small Vessel Diseases] explode all trees | 355 |
| #6 | MeSH descriptor: [Intracranial Arterial Diseases] explode all trees | 1721 |
| #7 | MeSH descriptor: [Intracranial Embolism and Thrombosis] explode all trees | 448 |
| #8 | MeSH descriptor: [Intracranial Hemorrhages] explode all trees | 3113 |
| #9 | MeSH descriptor: [Stroke] this term only | 15636 |
| #10 | MeSH descriptor: [Brain Infarction] explode all trees | 1854 |
| #11 | MeSH descriptor: [Stroke, Lacunar] explode all trees | 70 |
| #12 | MeSH descriptor: [Vasospasm, Intracranial] explode all trees | 230 |
| #13 | MeSH descriptor: [Vertebral Artery Dissection] explode all trees | 10 |
| #14 | (stroke* OR poststroke OR (post NEXT stroke) OR apoplex* OR (cerebral NEXT vasc*) OR (brain NEXT vasc*) OR cerebrovasc*):ti,ab,kw (Word variations have been searched) | 82156 |
| #15 | ((brain* OR cerebr* OR cerebell* OR vertebrobasil* OR hemispher* OR intracran* OR (intra NEXT cran*) OR intracerebral OR infratentorial OR (infra NEXT tentorial) OR supratentorial OR (supra NEXT tentorial) OR (middle NEXT cerebral NEXT arter) OR MCA* OR (anterior NEXT circulation*) OR (posterior NEXT circulation*) OR (basilar NEXT arter*) OR (vertebral NEXT arter*) OR (space NEXT occupying) OR spaceoccupying) NEAR/2 (ischemi* OR ischaemi* OR infarct* OR thrombo* OR emboli* OR occlus* OR hypoxi*)):ti,ab,kw (Word variations have been searched) | 16590 |
| #16 | ((brain* OR cerebr* OR cerebell* OR intracerebral OR intracran* OR intra-cran* OR parenchymal OR intraparenchymal OR intraventricular OR (intra NEXT ventricular) OR infratentorial OR (infra NEXT tentorial) OR supratentorial OR (supra NEXT tentorial) OR (basal NEXT gangli*) OR putaminal OR putamen OR (posterior NEXT fossa) OR hemispher* OR subarachnoid) NEAR/2 (haemorrhage* OR hemorrhage* OR haematoma* OR hematoma* OR bleed*)):ti,ab,kw (Word variations have been searched) | 13918 |
| #17 | MeSH descriptor: [Hemiplegia] explode all trees | 0 |
| #18 | MeSH descriptor: [Paresis] explode all trees | 1101 |
| #19 | MeSH descriptor: [Gait Disorders, Neurologic] explode all trees | 981 |
| #20 | (hemipleg* OR hemipar* OR paresis OR paraparesis OR paretic):ti,ab,kw (Word variations have been searched) | 8538 |
| #21 | #1 OR #2 OR #3 OR #4 OR #5 OR #6 OR #7 OR #8 OR #9 OR #10 OR #11 OR #12 OR #13 OR #14 OR #15 OR #16 OR #17 OR #18 OR #19 OR #20 | 98742 |
| #22 | MeSH descriptor: [Social Cognition] this term only | 39 |
| #23 | MeSH descriptor: [Social Perception] explode all trees | 1100 |
| #24 | MeSH descriptor: [Facial Expression] explode all trees | 877 |
| #25 | MeSH descriptor: [Facial Recognition] explode all trees | 177 |
| #26 | MeSH descriptor: [Social Behavior] explode all trees | 10020 |
| #27 | MeSH descriptor: [Theory of Mind] explode all trees | 150 |
| #28 | MeSH descriptor: [Mentalization] explode all trees | 48 |
| #29 | MeSH descriptor: [Empathy] explode all trees | 884 |
| #30 | (social NEAR/2 cogn*):ti,ab,kw (Word variations have been searched) | 4408 |
| #31 | (social NEAR/2 percep*):ti,ab,kw (Word variations have been searched) | 1485 |
| #32 | (emotion* NEAR/2 percep*):ti,ab,kw (Word variations have been searched) | 338 |
| #33 | (emotion* NEAR/2 recogn*):ti,ab,kw | 835 |
| #34 | ((facial* OR face) NEAR/2 expression*):ti,ab,kw (Word variations have been searched) | 2037 |
| #35 | ((facial* OR face) NEAR/2 recogn*):ti,ab,kw (Word variations have been searched) | 816 |
| #36 | (social NEAR/2 behav*):ti,ab,kw (Word variations have been searched) | 5623 |
| #37 | (prosod* OR speech*):ti,ab,kw (Word variations have been searched) | 10071 |
| #38 | (theor* NEAR/2 (mind OR minds)):ti,ab,kw (Word variations have been searched) | 492 |
| #39 | (mentalising* OR mentalizing* OR mentalisation* OR mentalization* OR (ment* NEAR/2 process*)):ti,ab,kw (Word variations have been searched) | 1461 |
| #40 | ((pragmat* NEAR/2 impairment*)):ti,ab,kw (Word variations have been searched) | 15 |
| #41 | ((nonliteral* OR literal*) NEAR/2 language*):ti,ab,kw (Word variations have been searched) | 17 |
| #42 | (sarcasm* OR lie* OR joke*):ti,ab,kw (Word variations have been searched) | 6892 |
| #43 | (empath*):ti,ab,kw (Word variations have been searched) | 2839 |
| #44 | (perspect* NEAR/1 tak*):ti,ab,kw (Word variations have been searched) | 280 |
| #45 | #22 OR #23 OR #24 OR #25 OR #26 OR #27 OR #28 OR #29 OR #30 OR #31 OR #32 OR #33 OR #34 OR #35 OR #36 OR #37 OR #38 OR #39 OR #40 OR #41 OR #42 OR #43 OR #44 | 40158 |
| #46 | #21 AND #45 | 1971 |
| #47 | (clinicaltrials OR trialsearch):so | 490208 |
| #48 | (conference proceeding):pt | 236547 |
| #49 | #47 OR #48 | 726755 |
| #50 | #46 NOT #49 | 999 |
| **Limit search to Cochrane Reviews and Trials, and publication year 2000-2024** | | **858** |
| **MeSH descriptor: [] explode all trees** = term from the MeSH controlled vocabulary, including terms found below this term in the hierarchy **MeSH descriptor: [] this term only** = term from the MeSH controlled vocabulary, does not include terms found below this term in the MeSH hierarchy  **NEXT** = next to each other, in that specific order  **NEAR/1 / NEAR/2** = Next to each other, in any order, up to 1 / 2 word(s) in between  **:ti,ab,kw** = title, abstract and author keywords  **:pt** = publication type  **:so** = source  ***** = truncation of word for alternate endings | | |

##

Supplementary Table S3. Description of inclusion and exclusion criteria of articles included in the scoping review

| **Author (year of publication)** | **Exclusion criteria** | **Inclusion criteria** |
| --- | --- | --- |
| Aben et al. (2020) ^1^ | Severe cognitive disorder, pre-stroke dependence in ADL, life expectancy of <1 year, severe stroke, impossibility to participate in a neuropsychological assessment, an absolute contraindication to undergo an MRI-scan of the brain. | Aged 50 years and older, clinical diagnosis of acute stroke, evidence of cognitive dysfunction during hospitalization. |
| Adamaszek et al. (2014) ^2^ | History of psychiatric diagnosis, Mini Mental State Examination ≤ 26, Hamilton Rating Scale for Depression ≥ 10. | Cerebellar stroke, normal or corrected-normal vision. |
| Adamaszek et al. (2019) ^3^ | Concomitant neurologic or psychiatric history, Hamilton Rating Scale for Depression ≥ 10. | Cerebellar and supratentorial IS, normal or corrected vision, normal or corrected hearing. |
|  |  |  |
| Adams et al. (2020) ^4^ | Previous stroke/lesions, Transient Ischemic Attacks, ≥90 years, non-English speakers. | Not reported |
| Adams et al. (2021) ^5^ | Previous stroke, self-reported current diagnosis or history of any other neurological disorder or serious psychiatric illness. | MRI or CT confirmed first stroke, English proficiency, normal or corrected-to normal vision. |
| Alvarez-Fernandez et al. (2023) ^6^ | A history of head trauma, presence of moderate/severe depression, central nervous system disease, visual defect, any medical condition that could affect the cognitive performance, lacunar strokes, previous cerebrovascular pathology, territorial cerebral infarctions. | Age ≥18 years, ischemic lesion(s) after a single stroke, language preservation, and no history of previous cognitive impairment (as reported by the patient and/or family members). |
| Besharati et al. (2016) ^7^ | Previous history of neurological or psychiatric illness, ≤7 years of education, medication with significant cognitive or mood side-effects, language impairments that precluded completion of the study assessments. | Imaging-confirmed right-hemisphere lesion, contralateral hemiplegia, ≤4 months from symptom onset, right-handedness. |
| Blonder et al. (2012) ^8^ | A history of substance abuse, neurological disease other than RHD stroke, severe sensory impairment, and/or major medical or psychiatric comorbidities. | RHD stroke, right-handed, spoke English as a first language. |
| Braun et al. (2005) ^9^ | No history of further stroke, had psychiatric disorder or alcohol abuse, moderate or severe receptive aphasia, severe visual disturbance, severe internal disease, or severe cognitive deficits, the lesion in the brain stem. | First-event-stroke, IS or ICH. |
| Burke et al. (2020) ^10^ | Co-morbid, or previous neurological condition. | Clinical diagnosis of angiographically negative SAH, native English speakers. |
| Cheung et al. (2006) ^11^ | History of psychiatric, neurological diseases, alcohol abuse, head injury, or any other diseases known to affect the central nervous system. | First episode subcortical stroke, alert, normal eyesight, able to communicate appropriately, and able to follow instructions. |
| Cooper et al. (2014) ^12^ | N/A | Confirmed stroke diagnosis (symptoms >24 hours or positive CT scan) 1 year ago, no pre-existing neurological/psychiatric conditions or chronic drug/alcohol abuse, no severe cognitive impairment (MMSE score >23). |
| Cooper et al. (2015) ^13^ | Having a pre-existing neurological or psychiatric condition before a stroke, a history of chronic drug or alcohol abuse, severe cognitive impairment, indicated by a Mini-Mental State Examination score of 24 or lower, significant challenges in managing stroke recovery and rehabilitation. | A confirmed diagnosis of stroke, within 90 days of index stroke. |
| Corradi-Dell’Acqua et al. (2020) ^14^ | Evidence or history of previous neurological and psychiatric history. | A confirmed diagnosis of stroke. |
| de Souza et al. (2021) ^15^ | Comorbid neurological diseases, neurodegenerative diseases, psychiatric disorders, inflammatory and infectious diseases, altered level of consciousness (Glasgow Coma Scale ≤14), and aphasia at admission. | ≥18 years old at onset, ischemic stroke. |
| Dominguez et al. (2019) ^16^ | Premorbid history of neurological disease, including stroke, not having English as their first language, expressive dysphasia, and spatial neglect. | Focal lesions. |
| Ferreira Pereira et al. (2022) ^17^ | Cognitive impairment from causes other than stroke (e.g., mild cognitive impairment, dementia, Alzheimer’s), uncorrected visual or auditory disorders, hemineglect or hemianopsia, or unable to complete the assessment scales. | A clinical stroke diagnosis, be 18 or older, any sex, unilaterally affected, and meeting MMSE score criteria: ≥13 for illiterate, ≥18 for low/medium education, and ≥26 for higher education. |
| Hamilton et al. (2017) ^18^ | Non-native English speakers, inability to consent, medical history affecting cognition (e.g., autism, dementia), psychosis, aphasia, and uncorrected sensory impairments. | A first ever IS or ICH localized in one hemisphere within the past 6 months. |
| Harciarek et al. (2009) ^19^ | A history of cerebral disease or disorder prior to stroke, having mental retardation, psychiatric disorders, psychoactive drug treatment, or habitual drug or alcohol abuse. | IS in the right brain hemisphere. |
| Harciarek et al (2006) ^20^ | A history of cerebral disease or disorder prior to stroke, having mental retardation, psychiatric disorders, psychoactive drug treatment, or habitual drug or alcohol abuse. | IS limited only to the right hemisphere. |
| Jorna et al. (2021) ^21^ | Traumatic SAH | Aged 18 years or older, sufficient proficiency in the Dutch language, aneurysmal and angiographically negative SAH. |
| Kliszcz et al. (2004) ^22^ | N/A | Patients with IS in right hemisphere. |
| Kucharska-Pietura et al. (2003) ^23^ | Subjects with habitual drug or alcohol abuse, secondary neurological disorders, other psychiatric diagnoses, vision difficulties, or hearing issues. | A single episode cerebrovascular accident (IS or ICH), MMSE ≥ 23. |
| Luo et al. (2022) ^24^ | Coma, complications which may potentially affect emotion perception and recognition, history of previous stroke, history of depression, mental illness or congenital mental retardation, other neurological diseases, history of brain injury, severe hearing loss or vision loss, end-stage or other acute severe diseases, medications that may influence cognition, aphasia/dysarthria, or inability to complete neuropsychological tests, contraindication for MRI. | >18 years old, right-handed, MRI received within 48 hours of acute IS, MRI-evidenced acute IS in the left or right cerebral hemisphere, or infratentorial brain, NIHSS ≤ 10. |
| Nijsse et al. (2019) ^25^ | Having a condition interfere with study outcomes, dependent in daily activities before their stroke (Barthel Index ≤17), non-sufficient command of Dutch, cognitive decline (Heteroanamnesis List Cognition score ≤1), having visual neglect or language disorders. | A clinically and image confirmed diagnosis of stroke (IS or ICH), ≥18 years old. |
| Nijsse et al. (2019) ^26^ | Having a condition interfere with study outcomes, dependent in daily activities before their stroke (Barthel Index ≤17), non-sufficient command of Dutch, cognitive decline (Heteroanamnesis List Cognition score ≤1), having visual neglect or language disorders. | A clinically and image confirmed diagnosis of stroke (IS or ICH), ≥18 years old. |
| Pertz et al. (2022) ^27^ | Global aphasia or amnesia, severe neurological (other than stroke) or psychiatric illness currently or lifetime. | A confirmed diagnosis of IS/ICH, undergoing outpatient neuropsychological treatment. |
| Qu et al. (2022) ^28^ | A transient ischemic attack, lacked complete MRI data, showed no infarction on DWI, had hemorrhagic transformation complications, died during hospitalization, presented severe comorbidities, had a history of dementia or mental disorder, exhibited obvious cognitive dysfunction or severe depression upon admission, or if they or their relatives refused to provide written informed consent. | Age >18 years, a first acute IS occurring within the 7 days preceding admission, National Institute of Health stroke scale (NIHSS) score ≤15 on admission, a modified Rankin Scale score ≤2 on discharge. |
| Sensenbrenner et al. (2020) ^29^ | Severe aphasia, initial loss of consciousness, initial severe hemiparesis or hemiplegia, pre/post stroke dementia or mild cognitive disorder, vision or hearing loss, recurrent stroke from baseline to follow-up. | >18 years, first ever IS or ICH. |
| Sheppard et al. (2020) ^30^ | N/A | First stroke, premorbid fluent speakers of English, right-handed, did not have a neurologic disease other than stroke, did not have reduced level of consciousness or receive ongoing sedation, had normal or corrected-to-normal auditory and visual acuity based on neurologic examination. |
| Smith-Spijkerboer et al. (2022) ^31^ | Presence of psychiatric disease diagnosed, language barrier, evidence for a language disorder, previous neurological disorder, receiving rehabilitation, or inability to give informed consent. | ≥18 years, brain image verified IS, discharged directly home after hospital admission, minor stroke, defined as NIHSS ≤ 3p at discharge, modified Rankin Scale ≤ 2 at 6 weeks post-onset. |
| Souza et al. (2023) ^32^ | Having dementia, having cancer with neurological complications, individuals who died within the first year or were transferred to long-term care at discharge. | >18 years, brain image verified diagnosis of acute IS or ICH, first-ever stroke. |
| Stiekema et al. (2021) ^33^ | Serious conditions other than stroke that could affect study outcomes, evidence of visual neglect or serious aphasia, Barthel Index score ≤17 before stroke, ≥1 on the Hetero Anamnesis List Cognition test, insufficient command of the Dutch language. | Adults diagnosed with IS or ICH, confirmed by a CT scan in the acute phase. |
| Surian et al. (2001) ^34^ | History of cognitive impairment, psychiatric disorders, aphasia. | Unilateral damage due to stroke, single site lesion, fluent Italian. |
| Thomasson et al. (2021) ^35^ | Other than cerebellar stroke, diffuse and extensive white-matter disease, other degenerative or inflammatory brain disease, confusion or dementia, major psychiatric disease, the wearing of hearing aids or a history of tinnitus or a hearing impairment. | >18 years, cerebellar stroke, able to speak and understand French. |
| Thomasson et al. (2019) ^36^ | Other than cerebellar stroke, diffuse and extensive white-matter disease, other degenerative or inflammatory brain disease, confusion or dementia, major psychiatric disease, the wearing of hearing aids or a history of tinnitus or a hearing impairment. | >18 years, patients diagnosed with focal cerebellar lesions due to ischemic stroke, to speak and understand French. |
| Trippett et al. (2018) ^37^ | Prior neurological disease, reduced level of consciousness or ongoing sedation; uncorrected hearing/vision impairment, lack of premorbid competency in English, failure to follow task directions, not having MRI images. | Stroke in right hemisphere. |
| Tompkins et al. (2008) ^38^ | Bilateral lesions, brainstem or cerebellar damage, premorbid seizure disorders, head injuries requiring hospitalization, problems with drugs and/or alcohol, conditions such as Alzheimer’s or Parkinson’s disease, or psychiatric illness. | Unilateral hemispheric lesion confirmed by CT/MRI scan report, ≥4 months post-onset of CVA, ≥8 years of formal education. |
| Van den Berg et al.(2021) ^39^ | Serious psychiatric or neurological disorders, pre-existing cognitive decline, substance abuse. | IS in a cortical area of the cerebrum, understanding of the Dutch language. |
| Van den Berg et al. (2020) ^40^ | Lesions in the supratentorial region, possible dementia, possible depression or anxiety disorder, substance abuse, history of psychiatric or neurological disorders. | Isolated, cerebellar lesion verified via CT/MRI, understanding of the Dutch language. |
| Yeh et al. (2014) ^41^ | Any history of neurodegenerative disease, psychiatric disorder, moderate or severe depression, substance abuse, severe neuropsychological dysfunction. | Unilateral stroke. |
| Yip et al. (2004) ^42^ | Co-existing psychiatric illness, history of brain injury or any other neurological diseases. | Right-handed with sub-cortical lesions. |
| Buunk et al. (2016) ^43^ | <18 years, those with serious comorbidities, and those with insufficient proficiency in Dutch. | Nontraumatic SAH confirmed by CT scan. |
| Buunk et al. (2019) ^44^ | Insufficient proficiency of the Dutch language, serious co-morbidity. | SAH confirmed by CT in the acute stage, ≥18 years, able to undergo neuropsychological assessment, employed before SAH. |
| Buunk et al. (2017) ^45^ | Current or previous neurological conditions, psychiatric disorder, substance abuse, <18 years, insufficient proficiency in the Dutch language. | Diagnosis of aneurysmal aSAH confirmed by CT on admission, along with CT angiography and/or digital subtraction angiography to confirm the presence of a symptomatic intracranial aneurysm. |
| Kuttenreich et al. (2022) ^46^ | Children and adults with peripheral facial paresis, other neurological or psychological diseases, no ability to consent. | Adults (≥18 years) with or without unilateral central facial paresis after a stroke, normal or corrected visual and hearing ability, ability to consent. |
| Klepzig et al. (2023) ^47^ | ≥90 years, presence of cognitive deficits reported by attending physicians or relatives, history of schizophrenia, neurodegenerative disorders, epilepsy, brain traumas, or tumors. | >18 years, stroke ≥ 5 months, brain image verified stroke within the first week after stroke. |
| Tsolakopoulos et al. (2023) ^48^ | Psychiatric or neurological disorder other than stroke. | A single stroke lesion RBD, right-handedness, all were Greek native speakers. |
| Xi et al. (2013) ^49^ | Current, or previous psychiatric diagnoses, color blindness intelligence quotient, history of diffuse brain damage. | Unilateral temporal lobe IS, right-handed, and had normal or corrected-to-normal vision. |
| Leigh et al. (2013) ^50^ | Neurological disease other than stroke; reduced level of consciousness or on-going sedation; and inability to have MRI due to claustrophobia, implanted ferrous metal, or weight > 300 lb. | Patients with acute ischemic stroke in right hemisphere. |
| O'Connell et al. (2022) ^51^ | Bilateral stroke, incomplete data, significant comprehension difficulties. | Right-hemisphere stroke patients in the chronic phase, aged ≥45 years. |
| Pluta et al. (2017) ^52^ | Severe aphasia, unilateral spatial neglect confirmed in neuropsychological evaluation, and psychiatric disorders diagnosed by a medical doctor. | Native speakers of Polish, a single stroke, adult, sufficient cognitive functioning to participate in the study (e.g., capacity to give informed consent), and the ability to comprehend speech and produce long utterances. |
| Balaban et al. (2016) ^53^ | Left hemisphere stroke, severe cognitive impairment, stroke ≤ 2 months, lack of consent. | Stroke in right hemisphere, native Hebrew speakers, cortical or subcortical lesions. |
| Baldo et al. (2016) ^54^ | N/A | N/A |
| Humphreys et al. (2011) ^55^ | N/A | Patients with PPC/TPJ and frontal lobe lesions, all of whom had failed on pretests assessing ToM abilities. |
| Charbonneau et al. (2003) ^56^ | ICH, tumors, surgical lesions or encephalitis, confused or hospitalized in the interim or bedridden, prior/post-morbid psychiatric or toxicological history, severe language deficit, orofacial apraxia, deficit in the imitation or discrimination of non-emotional faces. | ≥12-month post-stroke, informed consent, ≥21 on MMSE. |
| Jospe et al. (2022) ^57^ | N/A | First-ever unilateral hemispheric stroke IS/ICH, no prior psychiatric or neurological issues, no signs of dementia or general brain atrophy, preserved core language functions at hospital discharge, >4 months after stroke, living at home. |
| Nakhutina et al. (2006) ^58^ | A history of neurological disease (other than stroke), substance abuse, psychiatric disorder, or mental retardation. | Right-handed, as assessed by a hand preference inventory, no reported history of converting from left-handedness, native speakers of English or fluent in English by 7 years of age. |
| Oishi et al. (2015) ^59^; | Reduced level of consciousness or ongoing sedation, neurological disease other than stroke, inability to have MRI. | Acute ischemic right hemisphere stroke, premorbid proficiency in English, provided informed consent to participate in the study, were able to complete the testing. |
| Thomasson et al. (2022) ^60^ | Brainstem or occipital lesion, multiple lesions, diffuse and extensive white matter disease, other degenerative or inflammatory brain diseases, confusion or dementia, major psychiatric disease, hearing aids, history of tinnitus or hearing impairment, agnosia, <18 years, major language comprehension deficits. | IS in cerebellum. |
| Thomasson et al. (2023) ^61^ | Brainstem or occipital lesion, additional brain lesions, diffuse and extensive white-matter disease, other degenerative or inflammatory brain diseases, confusion or dementia, major psychiatric disease, hearing impairments. | ≥18 years, first-ever cerebellar IS, chronic phase, French speakers. |
| Weed et al. (2010) ^62^ | Not reported. | Right hemisphere cortical lesion. |

ADL, activities of daily living; MRI, magnetic resonance imaging; RHD, rheumatic heart disease; IS, ischemic stroke; ICH, intracerebral hemorrhage; SAH, subarachnoid hemorrhage; NA, not applicable; CT, computed tomography; MMSE, ; NIHSS, National Institutes of Health Stroke Scale; CVA, cerebrovascular accident; aSAH, aneurysmal SAH; RBD, right brain damage; ToM, Theory of Mind; PPC, posterior parietal cortex; TPJ, temporoparietal junction

# References

1. Aben HP, Visser-Meily JM, Biessels GJ, de Kort PL, Spikman JM. High occurrence of impaired emotion recognition after ischemic stroke. *European Stroke Journal*. 2020;5:262-270. doi: <https://dx.doi.org/10.1177/2396987320918132>

2. Adamaszek M, D'Agata F, Kirkby KC, Trenner MU, Sehm B, Steele CJ, Berneiser J, Strecker K. Impairment of emotional facial expression and prosody discrimination due to ischemic cerebellar lesions. *Cerebellum*. 2014;13:338-345. doi: <https://dx.doi.org/10.1007/s12311-013-0537-0>

3. Adamaszek M, D'Agata F, Steele CJ, Sehm B, Schoppe C, Strecker K, Woldag H, Hummelsheim H, Kirkby KC. Comparison of visual and auditory emotion recognition in patients with cerebellar and Parkinson's disease. *Social Neuroscience*. 2019;14:195-207. doi: <https://dx.doi.org/10.1080/17470919.2018.1434089>

4. Adams AG, Henry JD, Molenberghs P, Robinson GA, Nott Z, von Hippel W. The relationship between social cognitive difficulties in the acute stages of stroke and later functional outcomes. *Social Neuroscience*. 2020;15:158-169. doi: <https://dx.doi.org/10.1080/17470919.2019.1668845>

5. Adams AG, Henry JD, von Hippel W, Laakso EL, Molenberghs P, Robinson GA, Schweitzer D. A comprehensive assessment of poststroke social cognitive function. *Neuropsychology*. 2021;35:556-567. doi: <https://dx.doi.org/10.1037/neu0000741>

6. Alvarez-Fernandez S, Andrade-Gonzalez N, Simal P, Matias-Guiu JA, Gomez-Escalonilla C, Rodriguez-Jimenez R, Stiles BJ, Lahera G. Emotional processing in patients with single brain damage in the right hemisphere. *BMC psychology*. 2023;11:8. doi: <https://dx.doi.org/10.1186/s40359-022-01033-x>

7. Besharati S, Forkel SJ, Kopelman M, Solms M, Jenkinson PM, Fotopoulou A. Mentalizing the body: spatial and social cognition in anosognosia for hemiplegia. *Brain*. 2016;139:971-985. doi: <https://dx.doi.org/10.1093/brain/awv390>

8. Blonder LX, Pettigrew LC, Kryscio RJ. Emotion recognition and marital satisfaction in stroke. *Journal of Clinical & Experimental Neuropsychology: Official Journal of the International Neuropsychological Society*. 2012;34:634-642. doi: <https://dx.doi.org/10.1080/13803395.2012.667069>

9. Braun M, Traue HC, Frisch S, Deighton RM, Kessler H. Emotion recognition in stroke patients with left and right hemispheric lesion: results with a new instrument-the FEEL Test. *Brain & Cognition*. 2005;58:193-201.

10. Burke T, Carr A, Loughnane A, Corr P, Nolan D, Coffey D, O'Hare A, Gillan D, Javadpour M, Pender N. Cognitive impairment in angiographically negative subarachnoid haemorrhage: A case-matched prospective study 1-year post-incident. *Cortex*. 2020;128:49-60. doi: <https://dx.doi.org/10.1016/j.cortex.2020.03.006>

11. Cheung CC, Lee TM, Yip JT, King KE, Li LS. The differential effects of thalamus and basal ganglia on facial emotion recognition. *Brain & Cognition*. 2006;61:262-268.

12. Cooper CL, Phillips LH, Johnston M, Radlak B, Hamilton S, McLeod MJ. Links between emotion perception and social participation restriction following stroke. *Brain Injury*. 2014;28:122-126. doi: <https://dx.doi.org/10.3109/02699052.2013.848379>

13. Cooper CL, Phillips LH, Johnston M, Whyte M, MacLeod MJ. The role of emotion regulation on social participation following stroke. *British Journal of Clinical Psychology*. 2015;54:181-199. doi: <https://dx.doi.org/10.1111/bjc.12068>

14. Corradi-Dell'Acqua C, Ronchi R, Thomasson M, Bernati T, Saj A, Vuilleumier P. Deficits in cognitive and affective theory of mind relate to dissociated lesion patterns in prefrontal and insular cortex. *Cortex*. 2020;128:218-233. doi: <https://dx.doi.org/10.1016/j.cortex.2020.03.019>

15. de Souza MFD, Cardoso MGF, Vieira ELM, Rocha NP, Vieira T, Pessoa AE, Pedroso VSP, Rachid MA, de Souza LC, Teixeira AL, et al. Clinical correlates of social cognition after an ischemic stroke: preliminary findings. *Dementia & Neuropsychologia*. 2021;15:223-229. doi: <https://dx.doi.org/10.1590/1980-57642021dn15-020010>

16. Dominguez DJ, Nott Z, Horne K, Prangley T, Adams AG, Henry JD, Molenberghs P. Structural and functional brain correlates of theory of mind impairment post-stroke. *Cortex*. 2019;121:427-442. doi: <https://dx.doi.org/10.1016/j.cortex.2019.09.017>

17. Ferreira Pereira NK, de Medeiros Cirne GN, de Oliveira Galvao FR, Costa ME, Dos Santos Lima Junior W, Azevedo Cacho EW, Mh NC, de Oliveira Cacho R. Reliability of the Theory of Mind Task Battery (ToM TB) to assess social cognition in post-stroke patients. *Topics in Stroke Rehabilitation*. 2022;29:499-506. doi: <https://dx.doi.org/10.1080/10749357.2021.1948155>

18. Hamilton J, Radlak B, Morris PG, Phillips LH. Theory of Mind and Executive Functioning Following Stroke. *Archives of Clinical Neuropsychology*. 2017;32:507-518. doi: <https://dx.doi.org/10.1093/arclin/acx035>

19. Harciarek M, Heilman KM. The contribution of anterior and posterior regions of the right hemisphere to the recognition of emotional faces. *Journal of Clinical & Experimental Neuropsychology: Official Journal of the International Neuropsychological Society*. 2009;31:322-330. doi: <https://dx.doi.org/10.1080/13803390802119930>

20. Harciarek M, Heilman KM, Jodzio K. Defective comprehension of emotional faces and prosody as a result of right hemisphere stroke: modality versus emotion-type specificity. *Journal of the International Neuropsychological Society*. 2006;12:774-781.

21. Jorna LS, Westerhof-Evers HJ, Khosdelazad S, Rakers SE, van der Naalt J, Groen RJM, Buunk AM, Spikman JM. Behaviors of Concern after Acquired Brain Injury: The Role of Negative Emotion Recognition and Anger Misattribution. *Journal of the International Neuropsychological Society*. 2021;27:1015-1023. doi: <https://dx.doi.org/10.1017/S135561772000140X>

22. Kliszcz J, Gąsecki D, Bandurski T, Nyka W. Emotional and cognitive empathy disturbances in right hemisphere - Ischemic stroke - Damaged patients SPECT imaging pilot study. *Polish Psychological Bulletin*. 2004;35:225-230.

23. Kucharska-Pietura K, Phillips ML, Gernand W, David AS. Perception of emotions from faces and voices following unilateral brain damage. *Neuropsychologia*. 2003;41:1082-1090.

24. Luo L, Huang X, Xiao Y, Chen R, Yu E, Yuan Q, Huang Y, Huang H, Chen X, Pan X. Facial emotion perception and recognition deficits in acute ischemic stroke. *Journal of Clinical Neuroscience*. 2022;106:219-225. doi: <https://dx.doi.org/10.1016/j.jocn.2022.10.002>

25. Nijsse B, Spikman JM, Visser-Meily JM, de Kort PL, van Heugten CM. Social Cognition Impairments in the Long Term Post Stroke. *Archives of Physical Medicine & Rehabilitation*. 2019;100:1300-1307. doi: <https://dx.doi.org/10.1016/j.apmr.2019.01.023>

26. Nijsse B, Spikman JM, Visser-Meily JMA, de Kort PLM, van Heugten CM. Social cognition impairments are associated with behavioural changes in the long term after stroke. *PLoS ONE [Electronic Resource]*. 2019;14:e0213725. doi: <https://dx.doi.org/10.1371/journal.pone.0213725>

27. Pertz M, Braunwarth JI, Steinbach J, Wising S, Thoma P. Social problem solving and trait socioemotional abilities in ambulatory stroke patients. *Journal of Clinical & Experimental Neuropsychology: Official Journal of the International Neuropsychological Society*. 2022;44:195-209. doi: <https://dx.doi.org/10.1080/13803395.2022.2101619>

28. Qu JF, Zhou YQ, Liu JF, Hu HH, Cheng WY, Lu ZH, Shi L, Luo YS, Zhao L, Chen YK. Right Cortical Infarction and a Reduction in Putamen Volume May Be Correlated with Empathy in Patients after Subacute Ischemic Stroke-A Multimodal Magnetic Resonance Imaging Study. *Journal of Clinical Medicine*. 2022;11:31. doi: <https://dx.doi.org/10.3390/jcm11154479>

29. Sensenbrenner B, Rouaud O, Graule-Petot A, Guillemin S, Piver A, Giroud M, Bejot Y, Jacquin-Piques A. High Prevalence of Social Cognition Disorders and Mild Cognitive Impairment Long Term After Stroke. *Alzheimer Disease & Associated Disorders*. 2020;34:72-78. doi: <https://dx.doi.org/10.1097/WAD.0000000000000355>

30. Sheppard SM, Keator LM, Breining BL, Wright AE, Saxena S, Tippett DC, Hillis AE. Right hemisphere ventral stream for emotional prosody identification: Evidence from acute stroke. *Neurology*. 2020;94:e1013-e1020. doi: <https://dx.doi.org/10.1212/WNL.0000000000008870>

31. Smith-Spijkerboer W, Meeske K, van der Palen JAM, den Hertog HM, Smeets-Schouten AS, van Hout M, Dorresteijn LDA. Impaired Visual Emotion Recognition After Minor Ischemic Stroke. *Archives of Physical Medicine & Rehabilitation*. 2022;103:958-963. doi: <https://dx.doi.org/10.1016/j.apmr.2021.10.024>

32. Souza FR, Sales M, Laporte LR, Melo A, Ribeiro N. Discharge outcomes as predictors of social participation in the community after a stroke: a cohort study. *International Journal of Rehabilitation Research*. 2023;46:325-330. doi: <https://dx.doi.org/10.1097/MRR.0000000000000599>

33. Stiekema APM, Nijsse B, de Kort PLM, Spikman JM, Visser-Meily JMA, van Heugten CM. The relationship between social cognition and participation in the long term after stroke. *Neuropsychological Rehabilitation*. 2021;31:278-292. doi: <https://dx.doi.org/10.1080/09602011.2019.1692670>

34. Surian L, Siegal M. Sources of performance on theory of mind tasks in right hemisphere-damaged patients. *Brain & Language*. 2001;78:224-232.

35. Thomasson M, Benis D, Saj A, Voruz P, Ronchi R, Grandjean D, Assal F, Peron J. Sensory contribution to vocal emotion deficit in patients with cerebellar stroke. *NeuroImage Clinical*. 2021;31:102690. doi: <https://dx.doi.org/10.1016/j.nicl.2021.102690>

36. Thomasson M, Saj A, Benis D, Grandjean D, Assal F, Peron J. Cerebellar contribution to vocal emotion decoding: Insights from stroke and neuroimaging. *Neuropsychologia*. 2019;132:107141. doi: <https://dx.doi.org/10.1016/j.neuropsychologia.2019.107141>

37. Tippett DC, Godin BR, Oishi K, Davis C, Gomez Y, Trupe LA, Kim EH, Hillis AE. Impaired Recognition of Emotional Faces after Stroke Involving Right Amygdala or Insula. *Seminars in Speech & Language*. 2018;39:87-100. doi: <https://dx.doi.org/10.1055/s-0037-1608859>

38. Tompkins CA, Scharp VL, Fassbinder W, Meigh KM, Armstrong EM. A different story on "Theory of Mind" deficit in adults with right hemisphere brain damage. *Aphasiology*. 2008;22:42-61.

39. van den Berg NS, de Haan EHF, Huitema RB, Spikman JM. The neural underpinnings of facial emotion recognition in ischemic stroke patients. *Journal of Neuropsychology*. 2021;15:516-532. doi: <https://dx.doi.org/10.1111/jnp.12240>

40. van den Berg NS, Huitema RB, Spikman JM, Luijckx GJ, de Haan EHF. Impairments in Emotion Recognition and Risk-Taking Behavior After Isolated, Cerebellar Stroke. *Cerebellum*. 2020;19:419-425. doi: <https://dx.doi.org/10.1007/s12311-020-01121-x>

41. Yeh ZT, Tsai CF. Impairment on theory of mind and empathy in patients with stroke. *Psychiatry & Clinical Neurosciences*. 2014;68:612-620. doi: <https://dx.doi.org/10.1111/pcn.12173>

42. Yip JT, Leung KK, Li LS, Lee TM. The role of sub-cortical brain structures in emotion recognition. *Brain Injury*. 2004;18:1209-1217.

43. Buunk AM, Groen RJM, Veenstra WS, Metzemaekers JDM, van der Hoeven JH, van Dijk JMC, Spikman JM. Cognitive deficits after aneurysmal and angiographically negative subarachnoid hemorrhage: Memory, attention, executive functioning, and emotion recognition. *Neuropsychology*. 2016;30:961-969. doi: <https://dx.doi.org/10.1037/neu0000296>

44. Buunk AM, Spikman JM, Metzemaekers JDM, van Dijk JMC, Groen RJM. Return to work after subarachnoid hemorrhage: The influence of cognitive deficits. *PLoS ONE [Electronic Resource]*. 2019;14:e0220972. doi: <https://dx.doi.org/10.1371/journal.pone.0220972>

45. Buunk AM, Spikman JM, Veenstra WS, van Laar PJ, Metzemaekers JDM, van Dijk JMC, Meiners LC, Groen RJM. Social cognition impairments after aneurysmal subarachnoid haemorrhage: Associations with deficits in interpersonal behaviour, apathy, and impaired self-awareness. *Neuropsychologia*. 2017;103:131-139. doi: <https://dx.doi.org/10.1016/j.neuropsychologia.2017.07.015>

46. Kuttenreich AM, von Piekartz H, Heim S. Is There a Difference in Facial Emotion Recognition after Stroke with vs. without Central Facial Paresis? *Diagnostics*. 2022;12:15. doi: <https://dx.doi.org/10.3390/diagnostics12071721>

47. Klepzig K, Domin M, Wendt J, von Sarnowski B, Lischke A, Hamm AO, Lotze M. Structural integrity of the insula and emotional facial recognition performance following stroke. *Brain Communications*. 2023;5:fcad144. doi: <https://dx.doi.org/10.1093/braincomms/fcad144>

48. Tsolakopoulos D, Kasselimis D, Laskaris N, Angelopoulou G, Papageorgiou G, Velonakis G, Varkanitsa M, Tountopoulou A, Vassilopoulou S, Goutsos D, et al. Exploring Pragmatic Deficits in Relation to Theory of Mind and Executive Functions: Evidence from Individuals with Right Hemisphere Stroke. *Brain Sciences*. 2023;13:29. doi: <https://dx.doi.org/10.3390/brainsci13101385>

49. Xi C, Zhu Y, Zhu C, Song D, Wang Y, Wang K. Deficit of theory of mind after temporal lobe cerebral infarction. *Behavioral & Brain Functions [Electronic Resource]: BBF*. 2013;9:15. doi: <https://dx.doi.org/10.1186/1744-9081-9-15>

50. Leigh R, Oishi K, Hsu J, Lindquist M, Gottesman RF, Jarso S, Crainiceanu C, Mori S, Hillis AE. Acute lesions that impair affective empathy. *Brain*. 2013;136:2539-2549. doi: <https://dx.doi.org/10.1093/brain/awt177>

51. O'Connell K, Marsh AA, Edwards DF, Dromerick AW, Seydell-Greenwald A. Emotion recognition impairments and social well-being following right-hemisphere stroke. *Neuropsychological Rehabilitation*. 2022;32:1337-1355. doi: <https://dx.doi.org/10.1080/09602011.2021.1888756>

52. Pluta A, Gawron N, Sobanska M, Wojcik AD, Lojek E. The nature of the relationship between neurocognition and theory of mind impairments in stroke patients. *Neuropsychology*. 2017;31:666-681. doi: <https://dx.doi.org/10.1037/neu0000379>

53. Balaban N, Friedmann N, Ziv M. Theory of mind impairment after right-hemisphere damage. *Aphasiology*. 2016;30(12):1399-1423. doi: <https://dx.doi.org/10.1080/02687038.2015.1137275>

54. Baldo JV, Kacinik NA, Moncrief A, Beghin F, Dronkers NF. You may now kiss the bride: Interpretation of social situations by individuals with right or left hemisphere injury. *NEUROPSYCHOLOGIA*. 2016;80:133-141. doi: 10.1016/j.neuropsychologia.2015.11.001

55. Humphreys GW, Bedford J. The relations between joint action and theory of mind: a neuropsychological analysis. *Experimental Brain Research*. 2011;211:357-369. doi: <https://dx.doi.org/10.1007/s00221-011-2643-x>

56. Charbonneau S, Scherzer BP, Aspirot D, Cohen H. Perception and production of facial and prosodic emotions by chronic CVA patients. *Neuropsychologia*. 2003;41:605-613.

57. Jospe K, Genzer S, Mansano L, Ong D, Zaki J, Soroker N, Perry A. Impaired empathic accuracy following damage to the left hemisphere. *Biological Psychology*. 2022;172:108380. doi: <https://dx.doi.org/10.1016/j.biopsycho.2022.108380>

58. Nakhutina L, Borod JC, Zgaljardic DJ. Posed prosodic emotional expression in unilateral stroke patients: recovery, lesion location, and emotional perception. *Archives of Clinical Neuropsychology*. 2006;21:1-13.

59. Oishi K, Faria AV, Hsu J, Tippett D, Mori S, Hillis AE. Critical role of the right uncinate fasciculus in emotional empathy. *Annals of Neurology*. 2015;77:68-74. doi: <https://dx.doi.org/10.1002/ana.24300>

60. Thomasson M, Benis D, Voruz P, Saj A, Verin M, Assal F, Grandjean D, Peron J. Crossed functional specialization between the basal ganglia and cerebellum during vocal emotion decoding: Insights from stroke and Parkinson's disease. *Cognitive, Affective & Behavioral Neuroscience*. 2022;22:1030-1043. doi: <https://dx.doi.org/10.3758/s13415-022-01000-4>

61. Thomasson M, Ceravolo L, Corradi-Dell'acqua C, Mantelli A, Saj A, Assal F, Grandjean D, Peron J. Dysfunctional cerebello-cerebral network associated with vocal emotion recognition impairments. *Cerebral Cortex Communications*. 2023;4(1) (no pagination). doi: <https://dx.doi.org/10.1093/texcom/tgad002>

62. Weed E, McGregor W, Feldbaek Nielsen J, Roepstorff A, Frith U. Theory of Mind in adults with right hemisphere damage: What's the story? *Brain & Language*. 2010;113:65-72. doi: <https://dx.doi.org/10.1016/j.bandl.2010.01.009>
